# Adenovectors encoding RSV-F protein induce durable and mucosal immunity in macaques after two intramuscular administrations

**DOI:** 10.1038/s41541-019-0150-4

**Published:** 2019-12-20

**Authors:** N. C. Salisch, A. Izquierdo Gil, D. N. Czapska-Casey, L. Vorthoren, J. Serroyen, J. Tolboom, E. Saeland, H. Schuitemaker, R. C. Zahn

**Affiliations:** 1 Janssen Vaccines & Prevention B.V., Leiden, The Netherlands; 2 Janssen R&D, Leiden, The Netherlands; 3 Present Address: ProQR Therapeutics, Leiden, The Netherlands

**Keywords:** Drug discovery, Medical research

## Abstract

Respiratory Syncytial Virus (RSV) can cause severe respiratory disease, yet a licensed vaccine is not available. We determined the immunogenicity of two homologous and one heterologous intramuscular prime-boost vaccination regimens using replication-incompetent adenoviral vectors of human serotype 26 and 35 (Ad26 and Ad35), expressing a prototype antigen based on the wild-type fusion (F) protein of RSV strain A2 in adult, RSV-naive cynomolgus macaques. All regimens induced substantial, boostable antibody responses that recognized the F protein in pre- and postfusion conformation, neutralized multiple strains of RSV, and persisted for at least 80 weeks. Vaccination induced durable systemic RSV-F-specific T-cell responses characterized mainly by CD4+ T cells expressing Th1-type cytokines, as well as RSV-F-specific CD4+ and CD8+ T cells, IgG, and IgA in the respiratory tract. Intramuscular immunization with Ad26 and 35 vectors thus is a promising approach for the development of an optimized RSV vaccine expected to induce long-lasting humoral and cellular immune responses that distribute systemically and to mucosal sites.

## Introduction

Respiratory Syncytial Virus (RSV), recently reclassified as a member of the pneumovirus family,^1^ causes annual recurrent epidemics of upper and lower respiratory tract infection (LRTI) in people of all ages, with young infants and the elderly being particularly susceptible to severe disease in part owing to limitations in immune function compared with adults.^2,3^ With 64 million cases estimated annually, RSV is the leading cause of LRTI in children under the age of 5, causing approximately three million hospitalizations and up to 199,000 deaths worldwide, most of them in developing countries.^2^ Natural infection with RSV induces only short-lived and incomplete protection,^4^ allowing reinfection with RSV to cause considerable morbidity and mortality also in the elderly. Approximately 5.5% of adults over the age of 65 are infected with RSV annually, leading to pneumonia in 10–20% and death in 8% of those hospitalized owing to RSV.^5–7^ At present, no treatment for RSV infection is available and prophylaxis is limited to palivizumab, a humanized monoclonal antibody specific for the RSV-F protein, which is licensed for prevention of severe LRTI in high-risk infants.^8^ However, high costs and the need for monthly administration limit its application.^9^

Despite the significant disease burden, no vaccine is currently licensed for human use. Vaccine development for infants faces the obstacle of safety concerns raised after the occurrence of enhanced respiratory disease (ERD) following natural RSV infection in RSV-naive infants that had received a poorly protective formalin-inactivated RSV (FI-RSV) vaccine in the 1960ies.^10,11^ Although the functional mechanism for FI-RSV-induced ERD has not been fully elucidated yet, there are indications that a T helper cell type 2 (Th2) biased response and/or immune complex deposition and complement activation in the lung owing to the formation of poorly protective antibody responses with limited functional capability play a role in its etiology.^12,13^ In elderly, ERD is not of concern, however several vaccines aimed to protect this population have been discontinued owing to missing signs of efficacy.^14^

Protective immunity against RSV infection appears to involve multiple arms of the immune system. An important role is attributed to serum-neutralizing antibodies against RSV envelope proteins, mostly the fusion (F) glycoprotein, which are inversely associated with the risk of reinfection,^15,16^ reduce the risk of RSV-associated illness,^17,18^ LRTIs,^19^ and hospitalizations in both children and elderly adults.^20,21^ Recently, it was shown that these neutralizing antibodies are predominantly prefusion F-specific.^22^ Systemic T-cell responses to RSV may additionally contribute to protective immunity against RSV disease in infants and in the elderly, although their role is less unequivocal.^23–26^ Mucosal IgA and IgG responses in the nose and lung may further act as a first barrier against RSV infection at the portal of viral entry.^21,27^ In addition, abundance of RSV-specific tissue-resident memory T cells in the lung correlates with reduced symptoms and viral load, implying their capacity to protect against severe respiratory viral disease when humoral immunity is insufficient.^28^ Based on these observations, we hypothesize that a protective RSV vaccine needs to elicit RSV-specific immunity that is characterized by high levels of functional antibodies and a Th1-biased T-cell response that distributes systemically and to mucosal sites. Vaccines based on recombinant, replication-incompetent vectors derived from human Adenovirus serotypes with low pre-existing immunity on a population level, such as serotype 26 or 35 (Ad26, Ad35),^29,30^ potently induce humoral and cellular, Th1-biased immune responses against various encoded antigens in animal models^31–33^ and in humans.^34–37^ In addition, Adenoviral vectors have a favorable safety profile in humans including infants,^38^ and can be readily manufactured in high quantities under GMP conditions,^39^ making them an attractive platform for an RSV vaccine.

In this study, we determined the immunogenicity of three intramuscular prime-boost vaccination regimens using Ad26 and Ad35 expressing the F protein of RSV strain A2 (RSV-FA2) as a prototype RSV antigen in cynomolgus macaques. Ad26.RSV.FA2 and Ad35.RSV.FA2, were previously shown to induce RSV-specific humoral and Th1-biased cellular immune responses in mice, and to be safe, cross-neutralizing and protective against challenge with homologous and heterologous RSV strains in the cotton rat challenge and ERD model.^40^ Here we show that in macaques, all three regimens induced substantial, boostable, cross-neutralizing, and long-lasting antibody titers, and T-cell responses characterized by expression of the Th1-type cytokines interferon-gamma (IFNγ), tumor necrosis factor-alpha (TNFα), and interleukin 2 (IL-2) that could be measured in peripheral blood and the respiratory tract, where protective immunity is particularly important to prevent RSV-mediated LRTI. The high, regimen-independent, and boostable immunogenicity of this RSV prototype vaccine demonstrates that vectors based on Ad26 and Ad35, even when applied intramuscularly and in a homologous regimen, are a promising platform for the development of an RSV vaccine and can be expected to induce long-lasting humoral and cellular immune responses.

## Results

### Ad26.RSV.FA2 and Ad35.RSV.FA2 prime-boost regimens induce durable antibody responses that recognize RSV-F in its pre- and postfusion conformation and neutralize multiple RSV strains

**Fig. 1:**
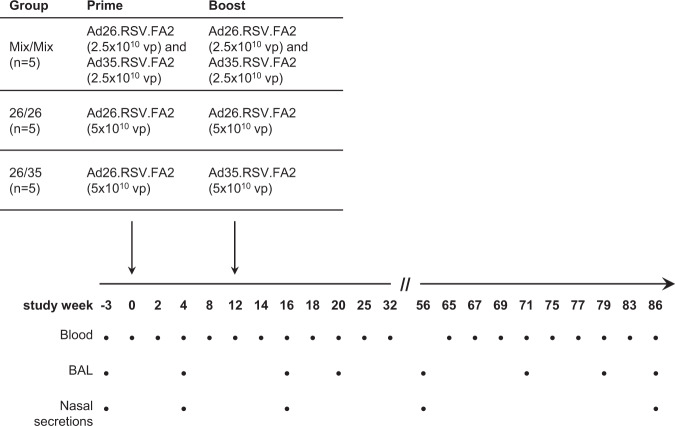
Immunization regimens, study timeline, and sampling time points. Filled circles represent a sampling time point.

Immune responses in cynomolgus macaques were measured over 86 weeks after intramuscular immunization with three prime-boost regimens using Ad26 and Ad35 expressing RSV-FA2. With an interval of 12 weeks, five animals each received either a homologous prime-boost of 2.5 × 10^10^ viral particles (vp) of Ad26.RSV.FA2 mixed with 2.5 × 10^10^ vp Ad35.RSV.FA2 (group “Mix/Mix”, 5 × 10^10^ vp total dose), a homologous prime-boost regimen of 5 × 10^10^ vp Ad26.RSV.FA2 (group “26/26”), or a heterologous prime-boost of 5 × 10^10^ vp Ad26.RSV.FA2 followed by 5 × 10^10^ vp of Ad35.RSV.FA2 (group “26/35”, see Fig. 1 for regimens, immunization timelines and sampling time points). Development of a neutralizing antibody response targeting the vector backbones was measured as an indication for vaccine take in the baseline Ad26- and Ad35-seronegative animals (Supplementary Fig. 1). Vector exposure was reflected in seroconversion and subsequent increases in Ad26-neutralizing antibody (NAb) titers in all animals receiving a prime and boost containing the Ad26-based vector (Supplementary Fig. 1A), whereas seroconversion upon prime immunization with Ad35 was rare (one of five animals) and re-exposure to the vector did not increase Ad35 NAb titers in all animals (Supplementary Fig. 1B), consistent with previous observations in NHPs and humans.^33,37^

**Fig. 2:**
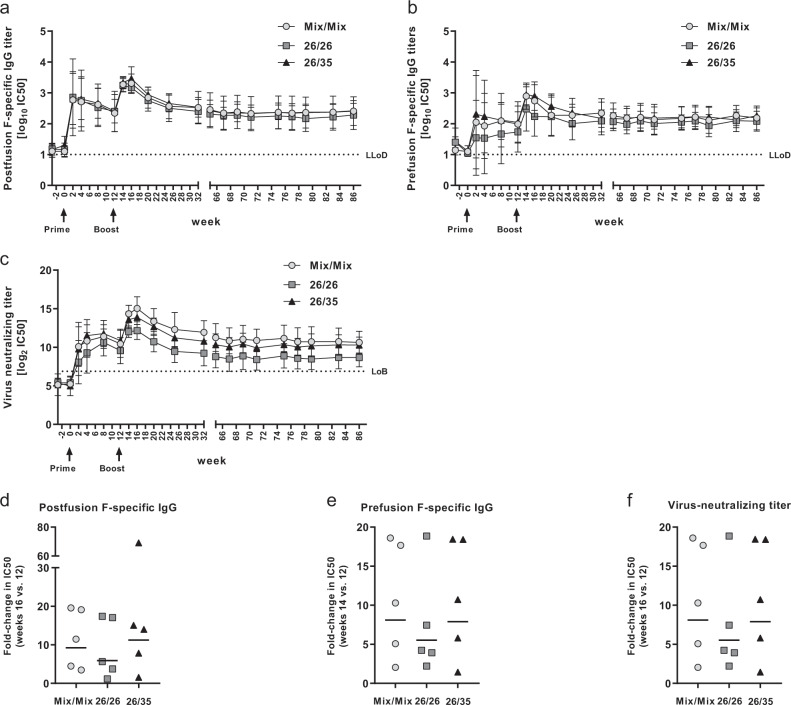
Humoral immune responses after immunization with the three RSV.FA2 vaccine regimens. **a**, **b** Binding antibody titers specific for RSV post-F **a** and pre-F **b** conformation. Serum antibody titers were measured by ELISA (*n* = 5 each for each regimen). Shown are arithmetic group means with standard deviation of the log10 inverse IC50 values determined relative to assay standard; LLoD = lower limit of detection (log_10_ lowest serum dilution in both the post-F and pre-F-specific assay, 1/10). **c** RSV-A2-neutralizing capacity. Neutralizing antibody titers were determined using an RSV-A2 based VNA (*n* = 5 each for each regimen). Shown are arithmetic group means with standard deviation of the log2 inverse IC50 values; LoB = limit of background (log_2_ 95th percentile of mean serum titers in all animals at all pre-immunization time points). **d–f** Fold-changes of humoral immune responses after boost. Shown is the ratio of binding antibody titers to post-F **d**, pre- **e**, and RSV-A2 virus neutralization titers **f** at the peak of the responses post boost (week 16 for post-F-specific IgG and virus neutralization titers, week 14 for pre-F-specific IgG) over those measured at the time of boost immunization at week 12. Fold-increases were calculated as a ratio of linear titers at week 16 (post-F ELISA and VNA) or week 14 (pre-F ELISA) divided by those at week 12. Each symbol represents one animal, bars depict geometric group means.

Although nonhuman primates (NHPs) are susceptible to RSV infection under experimental conditions,^41,42^ none of the animals in this study showed detectable serum antibody responses to the RSV-F protein prior to vaccination at week 0, or against the RSV-G protein at weeks 25, 32, and 65 (Fig. 2a, b, and Supplementary Fig. 2) as measured by enzyme-linked immunosorbent assay (ELISA), indicating that the animals were RSV inexperienced and did not get exposed to the virus after vaccination. Prime immunization with 5 × 10^10^ vp of the Ad26/Ad35 mix or Ad26 alone induced measurable antibody responses binding to RSV-F in its postfusion and prefusion conformations (post-F and pre-F) in all animals (Fig. 2a, b). No significant differences were detected between the three regimens in the mean post prime responses averaged over weeks 0–12 to the post-F (Mix/Mix 2.31 standard deviation (SD) 0.89, 26/26 2.34 SD 0.98, 26/35 2.40 SD 0.77) or pre-F proteins (Mix/Mix 1.89 SD 0.91, 26/26 1.51 SD 0.87, 26/35 1.96 SD 0.98; all *p* values > 0.05; ANOVA with post hoc Tukey correction for multiple comparisons), even though the mean pre-F-specific titers in the 26/26 group were slightly lower than those in the 26/35 group. Boost immunization with 5 × 10^10^ vp of either the Ad26/Ad35 mix, Ad26, or Ad35, induced a strong expansion of antibody responses to both post-F and pre-F in all animals, resulting in a peak 4 weeks post boost for post F-specific antibodies (Fig. 2a, d) and 2 weeks post boost for pre-F-specific antibodies (Fig. 2b, e). There were no significant differences between the three regimens in mean responses over weeks 14–32 to post-F (Mix/Mix 2.90 SD 0.48, 26/26 2.81 SD 0.46, 26/35 2.98 SD 0.46 SD) or pre-F (Mix/Mix 2.50 SD 0.53, 26/26 2.21 SD 0.56, 26/35 2.58 SD 0.51 and Fig. 2a, b; all *p* values > 0.05, ANOVA with post hoc Tukey correction for multiple comparisons). All three regimens induced similar mean fold-changes in the post-F and pre-F responses (Fig. 2d, e), with stronger individual fold-changes in animals that had lower pre-boost titers than in those with higher pre-boost titers. In all three groups, antibody titers against post- and pre-F contracted slightly by week 32, then remained at or above post prime levels until week 86, with no significant differences detected (averaged over weeks 66–86 for post-F: Mix/Mix 2.37 SD 0.48, 26/26 2.25 SD 0.39, 26/35 2.37 SD 0.21 SD; for pre-F: Mix/Mix 2.19 SD 0.37, 26/26 2.04 SD 0.29, 26/35 2.19 SD 0.30; all *p* values > 0.05, ANOVA with post hoc Tukey correction for multiple comparisons).

In all animals, RSV-F-specific antibodies raised by priming with either the Ad26/Ad35 mix or Ad26 alone neutralized the vaccine-homologous strain RSV-A2 in an assay based on the expression of Fluc from recombinant RSV-A2-like virus in target cells (Fig. 2c). In all groups virus neutralizing titers increased sharply 4 weeks post boost (Fig. 2c), with the Ad35/Ad26 Mix boost inducing an over 20-fold mean fold-change (mostly owing to one outlier) to a mean log2 IC50 titer of 15.3 1.5 SD, whereas boosting with Ad26 or Ad35 induced a more modest increase to 12.15 SD 1.16 for 26/26 and 13.87 SD 0.95 for 26/35 (Fig. 2f). Titers contracted slightly over time but remained at post-prime levels throughout the duration of the trial. No significant differences were detected between the three regimens post prime (averaged log2 IC50 titers over weeks 0–12: Mix/Mix 9.60 SD 2.82, 26/26 8.58 SD 2.62, 26/35 9.78 SD 3.21) and post boost (averaged log2 IC50 titers over weeks 14–32: Mix/Mix 13.39 SD 1.92, 26/26 10.71 SD 1.72, 26/35 12.42 SD 1.61), but during the long-term follow-up period titers in the 26/26 group dropped below those of the other two regimens, with the comparison with the Mix/Mix group reaching statistical significance (*p* = 0.011, averaged log2 IC50 titers over weeks 66–86: Mix/Mix 10.88 SD 1.57, 26/26 8.65 SD 1.35, 26/35 10.21 SD 1.14; ANOVA with post hoc Tukey correction for multiple comparisons). This may be owing to the limited sample size (*n* = 5 per group) and natural variation in the efficiency of the Ad26 prime immunization, as evidenced by the higher post-prime responses in the 26/35 group that was primed with the same vector.

The ratio of antibodies specific for post-F and pre-F slightly favored post-F in the weeks immediately following prime and boost immunization in all three regimens (Supplementary Fig. 3A), whereas a more balanced pre-/post-F-specific antibody response was reached during the long-term follow-up period. Although pre-F-specific antibodies have been described as the predominant mediators of viral neutralization,^22,43–45^ in this study viral neutralization titers were positively correlated with both the pre- and post-F-specific binding antibody titers in data pooled across the three regimens and over all time points post-prime (week 2–86; Spearman correlation: *r* = 0.7283 for post-F and *r* = 0.6855 for pre-F-specific IgG, *n* = 270; Supplementary Fig. 3B, C). Finally, cross-neutralization of RSV A strains A 11-050878, A 13-0000323, A 18-0011989, A CL57 and RSV-B strains RSV-B 11-052099, B 14-006938, B 17-058221, and B1 was determined by neutralization assays in sera pooled by vaccination group at baseline, week 16, and week 86 (Supplementary Fig. 4) and found to be of comparable magnitude across strains and durable until the end of the observation period.

### RSV-F-specific IFNγ ELISPOT responses persist for over 1 year after immunization with Adenoviral vectors expressing RSV-FA2

**Fig. 3:**
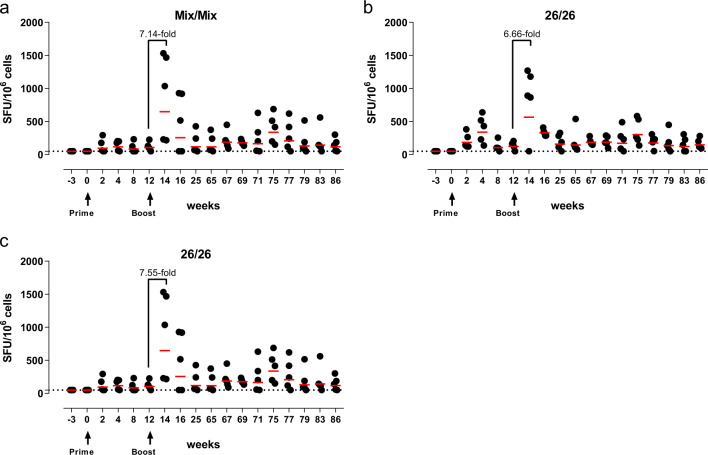
RSV-F-specific IFNγ ELISPOT responses. IFNγ ELISPOT responses in PBMC were determined after stimulation with a pool of 15-mer peptides overlapping by 11 amino acids, covering the RSV-F protein sequence. **a**–**c** Shown are background subtracted values per animal receiving a homologous prime-boost with the Ad26/Ad35 mix **a**, a homologous prime-boost with Ad26 **b**, or a heterologous prime-boost with Ad26 and Ad35 **c**. Dotted line = assay threshold based on CV, at 50 SFU/10^6^ cells. Red bars depict geometric group means.

RSV-F-specific T-cell responses were assessed longitudinally in blood using an IFNγ enzyme-linked immunospot (ELISPOT; Fig. 3). RSV-F-specific IFNγ ELISPOT responses were detectable within 2 weeks post-prime in the Mix/Mix (2/5 animals) and the 26/26 groups (all animals) (Fig. 3a–c). In all three groups, boost immunization induced a substantial, more than sixfold expansion of the mean IFNγ ELISPOT response (7.14-fold in Mix/Mix group, 6.66-fold in the 26/26 group, 7.55-fold in the 26/35 group) within 2 weeks post boost. There were no significant differences between groups in the ratio of peak post (week 14) over pre-boost (week 12) responses (all *p* values > 0.05; ANOVA for potentially censored values (Tobit analysis) with post hoc Tukey correction for multiple comparisons). Two animals in the Mix/Mix group showed low post boost responses, corresponding with low RSV-F-specific humoral immune responses throughout (all readouts). In all groups, IFNγ ELISPOT responses contracted to levels at or above those detected post-prime by week 25 and persisted at those levels until week 86.

### Systemic T-cell responses are mainly mediated by CD4+ T cells that express TNFα or IL-2 in addition to IFNγ

**Fig. 4:**
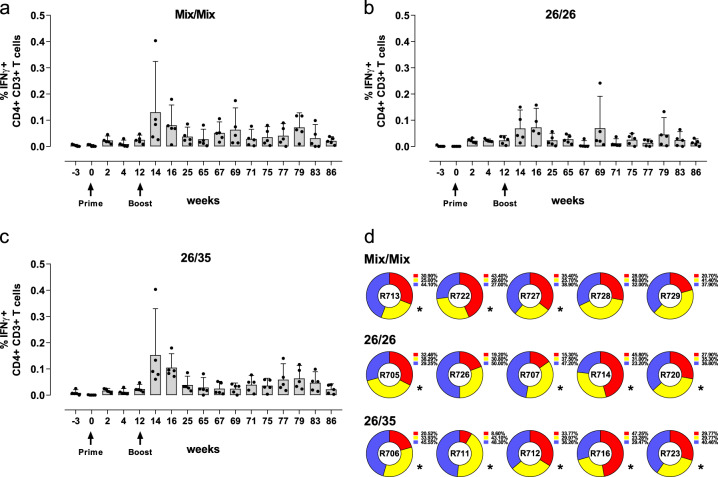
RSV-F-specific ICS responses in PBMC and polyfunctionality post boost. RSV-F-specific IFNγ responses were determined in peripheral CD4+ T cells using polychromatic flow cytometry after stimulation with RSV-F peptides. **a–c** Shown are arithmetic group means of IFNγ expression in CD4+ T cells with upper 95% confidence limit (upper whisker) of individually background subtracted values. Animals received a homologous prime-boost with the Ad26/Ad35 mix **a**, a homologous prime-boost with Ad26 **b**, or a heterologous prime-boost with Ad26 and Ad35 **c**. Arrows indicate immunization time points. The pie charts below summarize the polyfunctional composition of the cytokine response in individual animals two weeks post boost (study week 14). Functional subsets were determined by Boolean gating of IFNγ, TNFα, and IL-2 expressed after RSV-F peptide stimulation and each slice represents the fraction of the total RSV-F-specific cytokine response that consists of T cells expressing all three functional markers (red), any combination of two functional markers (yellow), or individual functional markers (gray). Positive responses to RSV-F peptide stimulation within each functional T-cell population were determined per animal in comparison to the corresponding negative control, using a one-sided Fisher’s exact test with Bonferroni adjustment for multiple comparisons. If peptide stimulation induced a significant response for at least one cytokine, the animal is considered a responder, marked by and asterisk under the pie in this figure.

To further characterize qualitative aspects of the systemic T-cell response to the three vaccination regimens, RSV-F-specific IFNγ, TNFα, and IL-2 responses in CD4+ and CD8+ T cells were assessed longitudinally by intracellular cytokine staining (ICS) and polychromatic flow cytometry (see Supplementary Fig. 5 for gating strategy). Although systemic CD8+ T-cell responses were very low (data not shown), all three regimens induced RSV-F-specific IFNγ, TNFα, and IL-2 responses in CD4+ T cells upon prime. As for IFNγ ELISPOT responses, ICS responses increased upon boost immunization, peaking 2 weeks post boost (week 14) and remaining above background levels until the end of the observation period (IFNγ responses shown in Fig. 4a–c, TNFα, and IL-2 responses see Supplementary Fig. 6). No statistically significant differences were detected between the mean IFNγ, TNFα, or IL-2 responses per group after prime (week 2) or post boost (week 14; ANOVA for potentially censored values (Tobit analysis) with post hoc Tukey correction for multiple comparisons). A functional subset analysis on the peak response at week 14 further showed a high degree of polyfunctionality across all three groups, in which more than half of the RSV-F-specific CD4+ T cells in each individual animal produced all three or any combination of two cytokines upon stimulation (Fig. 4a–c, pie-graphs). A stringent, exploratory statistical responder analysis was performed at peak response on week 14, determining whether the antigen-specific T-cell responses after peptide stimulation differed significantly from background responses (one-sided Fisher’s exact test with Bonferroni adjustment for multiple comparisons). The analysis demonstrated a post boost responder rate of 100% for the 26/35 regimen (5/5 animals), followed by 80% in the 26/26 group (4/5 animals), and 60% in the Mix/Mix group (3/5 animals).

### Adenoviral vectors expressing RSV.FA2 induce strong cellular responses in the lung, consisting of CD8+ and CD4+ T cells with a high degree of polyfunctionality

**Fig. 5:**
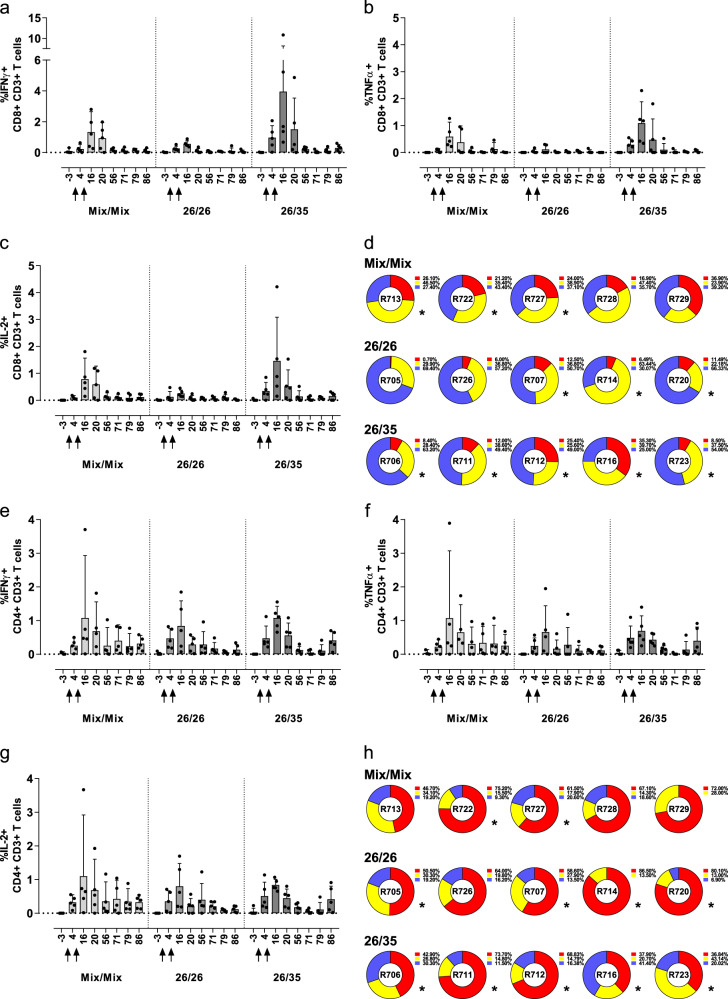
RSV-F-specific ICS responses in the lung and polyfunctionality post boost. RSV-F-specific CD8+ T cells **a**–**d** and CD4+ T cells **e–h** expressing IFNγ **a**, **e**, TNFα **b**, **f**, or IL-2 **c**, **g** were determined in cells extracted from BAL, using polychromatic flow cytometry, after stimulation with a pool of 15-mer peptides overlapping by 11 amino acids, covering the RSV-F protein sequence. Shown are arithmetic group means with upper 95% confidence limit (upper whisker) over time, of individually background subtracted values. Animals received a homologous prime-boost with the Ad26/Ad35 mix (light gray), a homologous prime-boost with Ad26 (dark gray), or a heterologous prime-boost with Ad26 and Ad35 (black). Arrows indicate immunization time points. Polyfunctional composition of the cytokine response in CD8+ T cells **e** and CD4+ T cells **h** of individual animals 4 weeks post boost (study week 16) was determined as described before and is summarized in the pie charts. Responders as defined by Fisher’s exact test are indicated by an asterisk. Data from individual animals in Figures **d**, **h** are shown in corresponding order.

To evaluate whether the different vaccine regimens induced mucosal immunity in the respiratory tract, RSV-F-specific responses were assessed in bronchoalveolar lavage (BAL) material at eight time points throughout the study (Fig. 1). RSV-F-specific cytokine responses were determined on CD4+ and CD8+ T cells in purified BAL cells by ICS. In contrast to the blood, all three regimens induced strong antigen-specific IFNγ, TNFα, and IL-2 CD8+ T-cell responses in the lung (Fig. 5a–c), which were detectable 4 weeks post-prime. Responses increased considerably after boost administration (week 16) and remained detectable until week 86. CD4+ T-cell responses, which were ~10-fold higher than those determined in the blood, followed the same kinetics. No significant differences were detected in the group mean CD4 + responses after prime (week 4) or after boost (week 16) for any of the three measured cytokines (ANOVA for potentially censored values (Tobit analysis) with post hoc Tukey correction for multiple comparisons). Within the CD8+ T-cell subset, lower responses for all three measured cytokines were observed in the 26/26 group compared with the 26/35 group (*p* = 0.007 for IFNγ, *p* = 0.0009 for TNFα, and *p* = 0.031 for IL-2) and lower TNFα responses compared to the Mix/Mix group (*p* = 0.0026) after boost (ANOVA for potentially censored values (Tobit analysis) with post hoc Tukey correction for multiple comparisons). A functional subset analysis performed on the peak boost response in the lung at week 16 revealed a high degree of polyfunctionality within the CD8+ and CD4+ T-cell compartments. A particularly high degree of polyfunctionality was detected in the CD4+ T-cell compartment, with more than half of the antigen-specific cells expressing all three evaluated cytokines in most animals, and any combination of two of them in a sizable percentage of the remaining cells (Fig. 5h). The polyfunctional profiles of the CD4+ and CD8+ T-cell responses were not statistically compared, but no obvious differences were detected between the three regimens. The antigen-specific CD8+ T cells showed a lower degree of tri-functionality compared with CD4+ T cells owing to lower expression of IL-2 as characteristic of an effector-type CD8+ T-cell response, but tri- and bi-functional T cells still constituted more than half of the antigen-specific CD8+ T-cell response in more than half of the animals (Fig. 5d). The exploratory statistical responder analysis reflected the high peak responses in the lung at week 16, demonstrating that—as in blood—100% of the animals in the 26/35 group were statistical responders within the CD4+ and the CD8+ T-cell compartments (Fig. 4c). High responder rates were also observed in the 26/26 and Mix/Mix groups.

To understand whether vaccine-induced T-cell responses in the periphery would be indicative of those induced in the lung, week 14 RSV-F-specific IFNγ ELISPOT and IFNγ + CD4+ T-cell ICS responses in blood were correlated with week 16 RSV-F-specific IFNγ + CD8+ and CD4+ T-cell responses in the lung, respectively, pooled across all three regimens (Supplementary Fig. 7). IFNγ ELISPOT responses in blood correlated moderately with IFNγ + CD8+ T-cell responses in the lung (Supplementary Fig. 7A; *r* = 0.5581, Spearman correlation) but weakly with the IFNγ + CD4+ T-cell response (Supplementary Fig. 7B; *r* = 0.3113, Spearman correlation), possibly stemming from the inherent differences between ELISPOT and ICS in defining the parental cell population. However, a strong correlation was detected between RSV-F-specific IFNγ + CD4+ T cells in blood and in the lung by the same assay (Supplementary Fig. 7C; *r* = 0.8571, Spearman; all correlations *n* = 15).

### Durable RSV pre-F-specific antibody responses are induced in the lung after immunization with adenoviral vectors expressing RSV.FA2

**Fig. 6:**
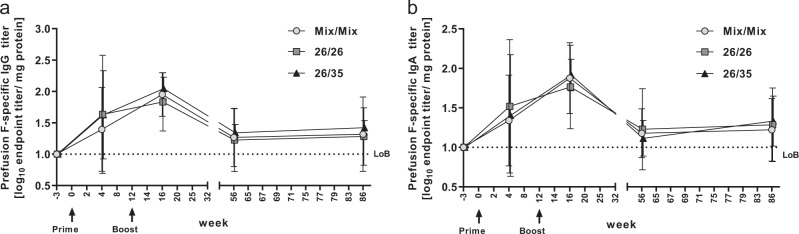
Pre-F RSV-specific IgG and IgA responses in BAL after immunization with the three RSV.FA2 vaccine regimens. Antibody titers were measured by IgG ELISA **a** and IgA ELISA **b** in BAL fluid obtained from animals immunized with the indicated regimens (*n* = 5 each) at the indicated time points. Shown are arithmetic group means with standard deviation of log-transformed endpoint titers, defined as the reciprocal dilution at which the signal reached a threshold set at 3× standard deviation above the mean signal of all samples at baseline. Individual titers per animal were normalized to the protein content of the BAL fluid as measured by OD280. LoB = limit of background, set to 1.

To assess whether the three regimens were able to induce vaccine-specific humoral immune responses in the respiratory mucosa, antibody responses specific for pre-F were measured by IgG and IgA ELISA in BAL fluid obtained at four time points after immunization. After prime, pre-F-specific IgG was detected in 9/15 animals across all groups (2/5 animals primed with Mix/Mix, 7/10 animals primed with Ad26, Fig. 6a). Titers peaked 4 weeks after boost, when all animals developed positive responses. By week 68, responses contracted but titers persisted until the end of the observation period, with 9/15 animals showing positive responses at week 86. Similar kinetics were observed for pre-F-specific IgA, with positive responses observed in 6/15 animals after prime (two animals from each group, Fig. 6b), in all animals at peak response 4 weeks after boost and in 8/15 animals at week 86. Pre-F-specific IgA responses were also measured in nasal secretions (Table 1), where all animals showed responses by week 16 (4 weeks after boost). By week 86, 13/15 animals still had detectable pre-F IgA titers in nasal secretions (2/5 animals in the Mix/Mix group had non-detectable titers at that time point). In both mucosal compartments, RSV-F-specific IgG and/or IgA titers were in the same order of magnitude for all three regimens and followed similar kinetics.

**Table 1:** Pre-F RSV-specific IgA titers in the nose.

	Week −3	Week 16	Week 86
Mix/Mix	0.200 (0.200–0.200)	1.406 (0.714–2.444)	0.464 (0.200–0.944)
26/26	0.220 (0.200–0.300)	0.967 (0.258–2.364)	0.938 (0.205–2.320)
26/35	0.200 (0.200–0.200)	1.407 (0.794–2.270)	1.218 (0.211–2.257)

Antibody titers were measured by IgA ELISA in nose swab eluates obtained from animals immunized with the indicated regimens (*n* = 5 each) at the indicated time points. Shown are arithmetic group means of log-transformed endpoint titers with ranges. Endpoint titer is defined as the reciprocal dilution at which the signal reached a threshold set at 3× standard deviation above the mean signal of all samples at baseline

Pre-F RSV-specific IgA titers in the nose

## Discussion

Three different intramuscular prime-boost immunization regimens with Ad26.RSV.FA2 and Ad35.RSV.FA2 induced strong and durable neutralizing antibody and T-cell responses to RSV-FA2 in cynomolgus macaques. Those responses were also present in the lung and nose, where protective immunity is particularly important to prevent RSV-mediated LRTI, and antibodies neutralized a wide array of RSV strains. All regimens induced immune responses of similar magnitude and quality across the assessed RSV-specific immune readouts, irrespective of whether a homologous or heterologous approach was chosen. This confirms our results in mice and cotton rats^40^ and indicates that simple intramuscular prime-boost regimens using Adenoviral vectors are a robust approach to generating strong, cross-neutralizing and durable vaccine-mediated RSV-specific immunity that is able to migrate to the respiratory tract. As adult cynomolgus macaques are only semi-permissive to RSV without showing any clinical signs of disease^46^ protective efficacy of the regimens was not assessed in this model. However, we previously demonstrated robust protective capacity of these vaccines against RSV-A2 and RSV B15/9 challenge in the permissive cotton rat model, compared with viral pre-exposure.^40^

Although post-F-specific antibodies are able to neutralize RSV in in vitro assays to a certain extent, most neutralizing antibodies in serum from RSV-exposed subjects target the pre-F protein.^22,44^ Pre-F-specific antibodies are reported to have higher affinity compared with those targeting post-F^45^ and vaccines using stabilized pre-F induced substantially higher neutralizing antibody titers than post-F in preclinical animal models.^47–49^ Wild-type F protein is intrinsically prone to refold from the labile pre- into the more stable post-F conformation on viral surfaces and in solution.^50^ We therefore hypothesize that by expressing the wild-type F protein in cells after transduction, both the prefusion and postfusion conformations are expected to be presented on the surface of the vaccine-transduced cells, allowing the induction of prefusion F- and postfusion F-specific antibodies. We indeed observed that the vaccine regimens induced pre- and post-F-specific antibodies with similar kinetics, but with a bias towards post-F targeting responses immediately after each vaccination in most animals (Supplementary Fig. 3A–C), which could confirm this hypothesis. Alternatively, antibodies recognizing epitopes common between pre- and post-F, which are detected by both used assays, may be induced more quickly. Over time, the ratio of pre- to post-F-specific antibody titers balanced, indicating that the initially high post-F-specific antibody responses may have been easier to induce, yet more short-lived than pre-F-specific responses, perhaps owing to different dynamics of B cell populations. Affinity maturation processes, on the other hand, likely play a role in the comparatively late onset of the pre-F-specific and neutralizing antibody responses.

To our knowledge, our study provides the longest preclinical follow-up of RSV-specific immunity upon vaccination to date, with durability being an important aspect of a vaccine intended to provide protection across annually recurring RSV epidemics. In contrast to reports on other vaccination approaches in NHPs, which either demonstrated rapid decline of initially high responses,^51^ did not assess durability,^52,53^ or applied a late boost immunization after set-point titers were reached,^51,54^ we established that prime-boost vaccination with Adenoviral vectors induced humoral and cellular RSV-specific immunity that persisted systemically and in the lung for more than a year and a half. This long-term durability in the absence of a late boost may be facilitated by the sustained, polyfunctional RSV-specific CD4+ T-cell responses induced upon vaccination, providing IL-2 mediated T-cell help over time.

Although the magnitude and polyfunctional cytokine profile of T-cell responses elicited by RSV vaccines based on unadjuvanted soluble protein or DNA have been shown to be rather modest,^51,54^ heterologous prime-boost regimens including a mucosal or intramuscular Adenoviral vector prime or boost have proven to induce higher and more polyfunctional systemic RSV-F-specific T-cell responses also in the absence of an adjuvant.^51,52^ Confirming and extending these findings, all three regimens in this study induced measurable systemic and mucosal T-cell responses upon prime and potently expanded them upon boost vaccination, including the homologous regimens. Systemic responses largely consisted of CD4+ T cells, as previously observed in NHPs vaccinated with Ad35-based vaccine regimens,^55^ but contrasts with reports of peripheral CD4+ and CD8+ T cells after vaccination of cynomolgus macaques with heterologous PanAd3 and modified vaccinia Ankara (MVA) regimens.^52^ In the lung, however, both CD8+ and CD4+ T-cell responses were induced that strongly exceeded the peripheral response. Although being ~ 10-fold higher, the frequency of RSV-specific CD4+ T-cell responses in the lung correlated well with the systemic responses using the same assay, which may indicate a biological link between systemic and mucosal T-cell responses after Adenoviral vaccination. To our knowledge RSV-F-specific BAL responses in the same order of magnitude have so far not been described after intramuscular immunization but could only be generated using complex combinations of systemic and mucosal vaccination with heterologous vaccine modalities. BAL responses exceeding systemic responses were for example observed in NHPs receiving a DNA prime by intramuscular electroporation followed by a heterologous, intra-tonsillar Adenoviral boost^51^ or in mice receiving a PanAd3-based intramuscular or intranasal prime, followed by a heterologous, intramuscular boost with MVA.^52^

Even though the limited size of the study precludes conclusions on any subtle qualitative differences between regimens, a generally high degree of polyfunctionality was detected in the antigen-specific CD4+ T-cell response in the periphery, and in CD4+ and CD8+ T cells in the lung at the post boost peak response for all three regimens. No non-Adenoviral comparator platform was included in this study, but a similarly high degree of polyfunctionality was observed after heterologous intramuscular electroporation reported above by Grunwald et al.,^41^, but not after a homologous intramuscular electroporation DNA/DNA regimen.^51^ Whether the induction of strong, polyfunctional antigen-specific T cells with the ability to migrate to the lung may be an inherent quality of Adenoviral vectored vaccines, irrespective of the route of administration remains to be confirmed. Contradicting previously observed insensitivity of the T-cell compartment to respond to repeated homologous administration of Adenoviral vectors,^51^ we observed expansion of RSV-F-specific immune responses, including T cells in blood and BAL, irrespective of whether the boost was heterologous (*n* = 5) or homologous (*n* = 10). Although boost immunization occurred in the presence of vector targeting immunity induced by the prime immunization, as evidenced by the presence of vector neutralizing antibodies (Supplementary Fig. 1), the responses induced by the homologous regimens did not consistently differ in magnitude, quality, or durability compared with the heterologous regimen, indicating that the negative impact of anti-vector immunity on vaccine immunogenicity was limited for these Ad26/Ad35-based vaccine regimens.

Recent assessments indicate that mucosal antibody responses may play a key role in protection of the respiratory tract against RSV infection. In an experimental controlled human infection model for RSV, pre-challenge nasal IgA titers against RSV were more clearly associated with protection than serum-neutralizing antibody titers.^27^ This emphasizes the importance of a potential RSV vaccine to induce mucosal and systemic RSV-specific antibody responses. Remarkably and unexpectedly, in this study intramuscular immunization with all three vaccine regimens induced durable RSV-F-specific antibody responses in the respiratory tract (in BAL and nasal secretions) of RSV seronegative animals.

The mechanisms of ERD have not been conclusively elucidated to date, but two aspects have been associated with vaccine-mediated ERD in preclinical models: a Th2-biased response and/or immune complex deposition and complement activation in the lung owing to the formation of poorly protective antibody responses with limited functional capability.^12,13^ The induction of Th2 cytokines was not assessed in the present study and animals were not exposed to an RSV challenge because adult cynomolgus macaques do not support productive infection with RSV.^42,56^ However, multiple findings indicate that the immune response induced by Ad26.RSV.FA2 and Ad35.RSV.FA2 used in this study is qualitatively distinct to responses predisposing towards ERD upon viral infection. First, in cynomolgus macaques, CD4+ and CD8+ responses in the lung were characterized by secretion of the Th1 cytokines IFNγ and TNFα. In addition, binding and functional antibodies as measured by ELISA and virus neutralizing assay (VNA), respectively, developed with similar, rather stable kinetics, indicating that there was no loss of functionality in the presence of a persistent binding antibody response. Finally, both vectors used in this study previously showed a strong Th1-type immune response in mice and did not induce ERD in the cotton rat model.^40^ Th1-type immunity has also been observed in response to an Ad26-based human immunodeficiency virus vaccine in humans^57^ and may be a characteristic intrinsic to the vector backbone irrespective of the encoded insert.^32^

In summary, we conclude that the largely regimen-independent, boostable, durable, and respiratory tract-directed immunogenicity of Ad26 and Ad35 vectors expressing the prototype antigen RSV-FA2 in the NHP model demonstrates that Adenoviral vectors are a promising vaccine platform for the development of an RSV vaccine for use in infants and elderly, expected to safely induce long-lasting humoral and cellular immune responses. The prototypic RSV-FA2 vaccines in this study have been assessed in two completed Ph1 studies in healthy adults (NCT02561871 and NCT02440035). Replacement of the prototypic wild-type FA2 antigen by a stabilized pre-F protein is expected to further enhance the magnitude and quality of the neutralizing antibody response induced by the Adenoviral vector platform, without changing systemic and mucosal responses. Therefore, ongoing clinical evaluation is focusing on optimized vectors expressing RSV-F in a stabilized pre-F conformation (studies NCT03334695, NCT03339713, NCT03303625, NCT02926430, NCT03606512, and NCT03502707).

## Materials and methods

### Ethics statement

The study was conducted in a facility assured by the NIH OLAW (National Institute of Health Office of Animal Welfare) and accredited by the USDA (United States Department of Agriculture) and AAALAC (International, Association for the Assessment and Accreditation of Laboratory Animal Care) operated by Advanced BioSience Laboratories Inc. (ABL, Rockville, MD; purchased by BIOQUAL Inc., Rockville, MD during conduct of the study, in February 2014). All animal research protocols were approved by the Institutional Animal Care and Use Committees (IACUC) of ABL and BIOQUAL, and the study was conducted in compliance with the Animal Welfare Act, Public Health Service Policy on Humane Care and Use of Laboratory Animals and other federal statutes and regulations relating to animals and experiments involving animals. Import and export permits for vectors and nonhuman primate (NHP) bio-specimens were obtained in compliance with US federal regulations and in accordance with the Convention on International Trade in Endangered Species of Wild Fauna and Flora (CITES) as overseen by the US Fish and Wildlife Service. All ethical regulations were complied with.

### Adenoviral vectors

Replication-incompetent, E1/E3-deleted recombinant adenoviral vectors based on adenovirus serotype 26 and serotype 35 were engineered using the AdVac system^31^ as described.^40^ A codon optimized, full-length gene encoding the fusion (F) protein from the RSV strain A2 (Genbank ACO83301.1) was inserted into the E1-position of the adenovirus genomes under transcriptional control of the human cytomegalovirus promoter and the SV-40 polyadenylation sequence. Cloning, rescue, and manufacturing of the replication deficient adenoviral vectors Ad26.RSV.FA2 and Ad35.RSV.FA2 in the complementing cell line PER.C6 was described in ref. ^31^ Virus particle (vp) titers in the viral preparations were quantified by measurement of optical density at 260 nm^58^ and infectivity was assessed by TCID50.^59,60^ The vp/IU ratio was between 1:1 and 6:1 for the viral preparations. Adenovirus-mediated expression of RSV-F was confirmed by western blot analysis of cell-culture lysates from infected A549 cells using a human monoclonal antibody against RSV-F (CR9503).

### Animals and housing

Five- to 6-year old, healthy female cynomolgus macaques (*Macaca fascicularis*) of Vietnamese origin (body weight 3.0–8.1 kg at study start) were purchased from Covance (Alice, TX) and kept in a BSL-2 facility under specific pathogen free conditions after screening negative for *Mycobacterium tuberculosis* (by Mantoux test), for simian immunodeficiency virus, simian retrovirus, and simian T-lymphotropic virus (all three by serology and polymerase chain reaction). Screening included Herpes B virus and measles serology, but results were not included in the SPF conditions. Animals were housed in stainless steel cages placed in study-dedicated, USDA-approved rooms under controlled, recorded environmental conditions of humidity, temperature and light (12-hour light cycle). Animals were pair-housed in groups of two or three animals of the same study group per cage except for brief, procedure-related periods, and provided sensory and cognitive environmental enrichment including manipulatable objects and foraging devices. Three times a day, animals were fed a standard NHP diet that consisted mainly of high-protein monkey biscuits, but included primatreats, soft dough diet, and a selection of fresh fruit, peanuts, cereals, or other treats. Tap water was provided ad libitum through an automated system. Animal well-being was monitored daily by husbandry staff and routine animal health surveillance, including blood chemistry and hematology, was provided by veterinary staff. Pre-set humane endpoints were used to define study-unrelated sacrifice criteria by a veterinarian. All measures were taken to minimize pain, distress and suffering and all procedures were performed by trained personnel. Upon termination of the study, animals were reassigned to other studies, no euthanasia was performed.

### Study design and animal procedures

All animal procedures were performed under anesthesia with either ketamine (10 mg/kg intramuscularly or intravenously), Dormitor (0.015 mg/kg intramuscularly), or under intubation with 3–5% isoflurane, depending on the length of the procedure. Using an online list randomizer (www.random.org), 15 animals were randomly assigned to three study groups of five animals each, receiving one of three prime-boost vaccination regimens (Fig. 1) with an interval of 12 weeks between prime and boost: a homologous prime-boost regimen with 5 × 10^10^ vp of a mixture of Ad26.RSV.FA2 and Ad35.RSV.FA2 (group “Mix/Mix”, 2.5 × 10^10^ vp of each serotype), or a homologous prime-boost with 5 × 10^10^ vp of Ad26.RSV.FA2 (group “26/26”), or a heterologous prime-boost with 5 × 10^10^ vp of Ad26.RSV.FA2 at prime and 5 × 10^10^ vp of Ad35.RSV.FA2 at boost (group “26/35”). No sample size calculation was performed, a sample size of *n* = 5 animals per group was chosen based on historical experience with Ad26-based vaccines in general, where this number provided an indication of humoral and cellular immunogenicity of vaccines in the NHP model. Blinding of the study was not possible owing to pair-housing of animals of the same study group. Animals were vaccinated intramuscularly in the quadriceps of the leg (0.5 ml/leg) with the indicated vector particle-doses in formulation buffer. Venous blood for peripheral blood mononuclear cells (PBMC) isolation or serum was collected from the femoral vein. Blood volumes taken did not exceed 12 ml/kg within 30 days and a maximum of 9 ml/kg at each individual bleeding time point. BAL was performed by inserting a premeasured, disposable plastic tube through a laryngoscope into the trachea using a guide wire, until the level of the manubrium. After removal of the guide wire, 50 ml of sterile d-PBS (Quality Biological, Gaithersburg, MD) was flushed into and immediately aspirated from the bronchus in installments of 10 ml at a time, using a syringe attached to the end of the plastic tube. BAL wash material was transferred into sterile 50 ml conical tubes and kept on ice until processing. Nasal swabs were gathered by inserting a pre-moistened sponge (Weck-Cel, Beaver-Visitec International, MA) into each naris, rotated in a continuous motion while entering deeper. The sponges were allowed to rest inside the naris for 5 min to absorb the nasal secretions, then placed into cryovials and frozen at − 70 °C.

### Processing of peripheral blood

Serum samples were prepared from clotted blood drawn into serum tubes after spinning at 1900 G for 5 min at room temperature (RT). Serum was stored at − 80 °C until time of analysis. PBMCs were isolated from whole blood drawn into anticoagulant-containing tubes (EDTA) by Ficoll density gradient centrifugation. Blood was diluted 1:1 with D-PBS without Ca_2_+ or Mg_2_+ (Quality Biological), underlayed with an equal volume of Ficoll-Paque Plus (GE Healthcare, Little Chalfont, UK) and spun at 1800 G for 20 min at RT. Buffy layers were transferred into a fresh tube, washed once with D-PBS, spun at 700 G for 10 min at RT, followed by lysis of residual red blood cells (RBCs) in RBC Lysis Solution (Qiagen, Hilden, Germany) for 10–15 min at RT. Lysis was stopped by addition of excess d-PBS and tubes spun at 1900 G for 5 min at RT. Viable cell numbers were subsequently determined by Trypan Blue exclusion using a Countess Automated Cell Counter (Thermo Fisher Scientific, Hampton, NH). Cells were adjusted to a concentration of 2 × 10^6^ cells/ml in RPMI-10 (RPMI complemented with 10% fetal bovine serum (Fisher Scientific, Hampton, NH), 10 mm Hepes buffer (Quality Biological, Gaithersburg, MD), 2 mm l-glutamine (Quality Biological), 100 μg/ml penicillin/streptomycin (Quality Biological) and kept on ice until analysis.

### Isolation of cells and fluids from BAL material

BAL wash fluid was strained through a 100 µm pore size cell strainer and spun at 700 G for 15 min at 4 °C. The supernatant (BAL fluid) was aliquoted and stored at − 80 °C until time of analysis. The cell pellet was visually inspected for the presence of red blood cells and if present, residual RBCs were lysed as described before. Viable cell numbers were determined as described before, then cell suspensions were adjusted to 2 × 10^6^ cells/ml in RPMI-10 and kept on ice until analysis.

### Isolation of fluids from nasal swabs

The swabs were incubated in 500 μl PBS with protease inhibitors (Roche, Basel, Switzerland) for 15 min at RT. The samples were transferred to a Nanosep 0.45 μm column (Sigma-Aldrich, St. Lois, MO) and centrifuged at 8000 G at 2–6 °C for 10 min. Fluid samples were stored at −20 °C.

### Determination of RSV-F-specific IgG and IgA in serum, nasal swabs, and BAL fluid by ELISA (pre-F, post-F)

Total serum IgG targeting pre-F and post-F were assessed by an ELISA qualified and validated for human sera. Twofold serial dilutions of individual, heat-inactivated (60 min at 57 °C) cynomolgus macaque serum samples in PBS with 1% FBS and 0.2% Tween20, starting at a 1:64 dilution were incubated for 2 h at RT in white LIA high-binding ELISA plates (Greiner Bio-One, Frickenhausen, Germany) that were either directly coated with a soluble RSV-post-F protein (F33, Janssen Vaccines, Leiden, Netherlands^49^) or first coated with streptavidin, followed by coating with biotinylated RSV-pre-F protein (SCTM, Janssen Vaccines, Leiden, NL, described in ref. ^49^). Those two proteins differ by five amino acids, which are not located in antibody epitopes. Reaction volumes were 100 µl. After washing in PBS with 0.5% Tween20, bound RSV-F-specific IgG was detected by adding 100 µl of a horseradish peroxidase (HRP)-conjugated, cross-reactive polyclonal mouse-α-human IgG Fcγ-specific antibody (Jackson ImmunoResearch Laboratories, West Grove, PA, catalog number 209-035-098) at a dilution of 1:3750. Conjugates were detected with a luminometric readout on a microplate reader (BioTek Synergy 4HT) with LumiGlo as a substrate. Inhibitory concentration 50 titers (IC50 titers) were determined using a 4-parameter logistic curve fit model using Gen5 software. Each serum sample was analyzed in duplicate. Pre-F-specific IgG in the lung was determined in crude BAL fluid using the ELISA conditions as described above, starting with BAL fluid diluted 1:1. Endpoint titers calculated as the reciprocal dilution at which the signal reached a threshold set at 3× standard deviation above the mean signal of samples collected at baseline. Titers were then normalized to the protein content of the individual BAL fluid samples as measured by OD280 and expressed as log10 endpoint titer per mg protein. Each BAL sample was analyzed in duplicate. Pre-F-specific IgA was measured in crude BAL fluid and nasal swab fluid as described above, using HRP-conjugated polyclonal goat anti-monkey IgA (Sigma-Aldrich, catalog number SAB3700759-1MG, 1:5000 dilution) instead of anti-human IgG. Endpoint titers were calculated as described for IgG measurements, including normalization to the protein content in the BAL.

### Determination of RSV-G-specific IgG in serum by ELISA

Total serum IgG targeting a type A central conserved domain G peptide (biotin-KQRQNKPPNKPNNDFHFEVFNFVPCSICSNNPTCWAICKRIPNKKPGKKTTTKPTKK sym-1705, Pepscan B.V., Lelystad, Netherlands) was assessed by ELISA. Fourfold serial dilutions of cynomolgus macaque serum samples in PBS with 1% FBS and 0.05% Tween20, starting at a 1:50 dilution were incubated for 1 h at RT in maxisorp ELISA plates (NUNC 439454, Thermo Fisher Scientific) that were first coated with avidin (400 ng/well in carbonate buffer), followed by coating with the biotinylated RSV-G peptide. Reaction volumes were 100 µl. After washing in PBS with 0.05% Tween20, bound RSV-G peptide specific IgG was detected by adding 100 µl of HRP-conjugated cross-reactive polyclonal mouse-α-human IgG Fcγ-specific antibody (Jackson ImmunoResearch Laboratories, catalog number 209-035-098) at a dilution of 1:3750. Conjugates were detected with OPD substrate (Thermo Fisher Scientific) at 492 nm absorbance readout (BioTek Powerwave 340, BioTek, Winooski, VT). Each serum sample was analyzed in duplicate. Endpoint titers were calculated as the reciprocal dilution at which the signal reached a threshold set at 1.5× the signal of naïve serum using linear interpolation.

### Adenoviral neutralization assay

Ad26 and Ad35 NAb titers in serum were assessed using a luciferase-based VNA based on the method described in ref. ^61^ A549 human lung carcinoma cells (catalog number ATCC CCL-185, obtained from the American Type Culture Collection, Manassas, VA) were plated at a density of 1 × 10^4^ cells/well in 96-well black-and-white isoplates (Wallac, Turku, Finland). E1/E3-deleted Ad26- or Ad35-luciferase reporter constructs were then added at a multiplicity of infection (MOI) of 500, together with twofold serial dilutions of individual heat-inactivated cynomolgus macaque serum samples starting at a 1:64 dilution for Ad26, or a 1:32 dilution for Ad35 in 200 μl reaction volumes. After incubation for 24 h at 37 °C and 10% CO_2_, luciferase activity in cryolysed cells was measured using the Neo-Lite Luciferase Assay System (PerkinElmer, Groningen, Netherlands) on a BioTek Synergy Neo luminescence counter (BioTek). Ninety percent neutralization titers (IC90) were defined as the maximum serum dilution that neutralized 90% of luciferase activity. Each serum sample was analyzed in duplicate.

### RSV neutralization assays

Titers of neutralizing antibodies targeting RSV-A2, RSV CL57, and RSV-B1 in serum were determined using a luciferase-based RSV VNA. Five thousand A549 human lung carcinoma cells (catalog number ATCC CCL-185, obtained from the American Type Culture Collection, Manassas, VA) were added to each well of 96-well white half-area plates (Greiner Bio-One, Frickenhausen, Germany) containing 2.5 × 10^4^ PFU/well of RSV-A2, RSV A CL57, or RSV-B1 viral particles encoding a luciferase reporter gene (resulting in an MOI of 5), together with threefold serial dilutions of individual heat-inactivated cynomolgus macaque serum samples starting at a 1:32 dilution in 100 μl reaction volumes. After incubation for 20 h at 37 °C and 10% CO_2_, luciferase activity in lysed cells was measured using the Neo-Lite Luciferase Assay System (PerkinElmer, Groningen, Netherlands) on a BioTek Synergy Neo luminescence counter (BioTek). Fifty percent neutralization titers (IC50) were defined as the maximum serum dilution that neutralized 50% of luciferase activity. Each serum sample was analyzed in duplicate. Titers of neutralizing antibodies targeting clinical isolates of RSV A, A 11-050878, A 13-0000323, A 18-0011989 and RSV-B, B 11-052099, B 14-006938, B 17-058221 (kindly provided by Dr. F.E.J. Coenjaerts, UMC Utrecht) were determined by a microneutralization assay. In brief, serially diluted heat-inactivated sera were mixed with 10,000 plaque-forming units (pfu) of virus in 96-well black & white flat bottom tissue culture plates and incubated for 1 h at RT. Subsequently 3 × 10^4^ VERO cells (WHO 10–87; 880101, obtained from WHO) per well were added and plates were incubated for 90 h at 37 °C, 10% CO_2_. The monolayers were washed and fixed with 80% cold acetone. RSV replication was determined by F protein expression with a biotin-conjugated anti-F monoclonal antibody (clone 133-1H, catalog number Mab8262B-5, Sigma-Aldrich) at a dilution of 1:1000 followed by Streptavidin-HRP (BD Pharmingen catalog number 5540667) at a dilution of 1:1000, and Lumiglo substrate. The luminescence signal was determined with the Biotek Synergy Neo luminescence counter (BioTek). VNA titers were calculated as the antibody concentration that caused a 50% reduction in luminescence, expressed as IC50 titers. Cell lines used in the neutralization assays were not additionally authenticated.

### IFNγ ELISPOT

RSV-FA2-specific, IFNγ-secreting T cells were enumerated in freshly isolated PBMCs using ELISPOT kit (Monkey IFNγ ELISpot^PRO^, MabTech, Cincinnati, OH) specific for monkey IFNγ. Plates pre-coated with an NHP IFNγ-specific capture antibody (clones GZ-4 or MT126L) were washed four times with sterile d-PBS (180 µl/well) and blocked with RPMI-10 (200 µl/well) for 30 min at 37 °C and 5% CO_2_. After removal of the blocking buffer, PBMCs in RPMI-10 were seeded at 2 × 10^5^ cells/well and stimulated with an RSV-FA2 peptide pool, reconstituted in dimethylsufoxide (DMSO), consisting of 15-mers overlapping by 11 amino acids at a final concentration of 2 µg/ml for 18–20 h at 37 °C and 5% CO_2_, in a final volume of 200 µl. RPMI-10 supplemented with 0.005% DMSO served as a medium control and a 1/1000 dilution of α-CD3 antibody in RPMI-10 as a positive control. After removal of the cell suspension, wells were washed five times with PBS + 0.05% Tween20 (KD Medical, Columbia, MD) and twice with D-PBS (200 µl/well) at RT and subsequently incubated with 100 µl/well INFγ detector antibody conjugated to alkaline phosphatase (clone 7-B6-1-ALP, 1:200 in PBS + 0.5% FBS) for 2 h at RT. Plates were washed as described before and spots developed for 15 min in the dark at RT, using 100 µl of a 5-bromo-4-chloro-3'-indolyphosphate p-toluidine/nitro-blue tetrazolium chloride solution filtered through a 0.45 µm filter. The development was stopped by washing extensively with tap water. Plates were air dried for at least 72 h before spots were counted on an ImmunoSpot S5 ELISPOT plate reader (C.T.L. Europe GmBH, Bonn, Germany). All samples were analyzed in either duplicates or triplicates. Mean spot-forming units (SFU) per 10^6^ cells were calculated from the replicate measurements, followed by individual background subtraction of the mean medium control values from the mean peptide-stimulated values. The lower limit of quantification was set at 50 SFU/10^6^ cells, which corresponds to a coefficient of variation of 30%.

### ICS

RSV-FA2-specific, IFNγ, TNFα, and IL-2-secreting CD8+ and CD4+ T cells were detected in freshly isolated PBMCs or BAL cells using multiparametric ICS and polychromatic flow cytometry, based on methods optimized for NHP cells as described.^62^ In total, 1.5 × 10^6^ PBMC, or 5 × 10^5^ BAL cells were stimulated overnight (12 h) at 37 °C with 2 µg/ml of an RSV-FA2 peptide pool consisting of 15-mers overlapping by 11 amino acids in the presence of 1 µg/ml soluble, fluorochrome-linked anti-CD28 antibody (BD Biosciences, clone L293, catalog number 337181) and 1 µl/ml brefeldin A (BD GolgiPlug) in 200 µl final volume of RPMI-10. Cells were stimulated in sterile 96-well round-bottom plates (VWR, Radnor, PA) coated with cross-linked, purified anti-CD49d (BD Biosciences, clone 9F10) catalog number 556634). PBMC aliquots kept in RPMI-10 with anti-CD28 (unstimulated) or incubated with 2.5 µg/ml phytohemagglutinin (Sigma-Aldrich) served as a negative and positive control, respectively. To coat plates with cross-linked co-stimulatory anti-CD49d, sterile 96-well round-bottom, tissue culture-treated polystyrene plates (VWR, Radnor, PA) were incubated overnight at 4 °C with 200 µl of 2.5 µg/ml affinity purified antibody F(ab’)2 fragments of Goat-anti-Mouse IgG(H + L) (Beckman Coulter, Carlsbad, CA, catalog number IM1618) in D-PBS, washed twice with D-PBS, incubated with 200 µl of 10 µg/ml of unconjugated, co-stimulating antibody targeting CD49d (BD Biosciences, clone 9F10, catalog number 556634) in D-PBS for ≥1 h at 37 °C, and washed one final time with D-PBS.

Stimulated cells were washed in FACS staining buffer (D-PBS with 0.5% FBS) and incubated in 200 µl 0.02% EDTA (UltraPure EDTA, 0.5 m. pH 8.0, Life Technologies, Carlsbad, CA) in D-PBS (Quality Biological) for 15 min at 37 °C to disengage interactions of the cells with the cross-linked co-stimulatory antibody. Subsequently, cells were washed in staining media, transferred into a 96 deep-well plate, and stained for 10 min at RT in the dark with 0.5 µl of a fixable Live/Dead cell discrimination fluorochrome (Life Technologies, Carlsbad, CA) in 1 ml. Cells were washed in FACS staining buffer, then surface stained for 30 min at RT in the dark with 100 µl of a cocktail containing antibodies targeting CD3 (BD Biosciences, clone SP34-2, catalog number 557917, 1 µl), CD4 (BD Biosciences, catalog number 562843, clone L200, 0.5 µl), CD8 (BD Biosciences, catalog number 557834, clone SK1, 5 µl), CD28 (BD Biosciences, clone L293, catalog number 337181, 20 µl), and CD95 (clone DX2, catalog number 561978, 20 µl). Cells were then washed in FACS staining buffer, fixed, and permeabilized with Caltag Fix & Perm solutions (Life Technologies), and incubated with 130 µl of a cocktail containing antibodies targeting CD69 (Beckman Coulter, clone TP1.55.3, catalog number 6607110, 5 µl), IL-2 (BD Biosciences, clone MQ1-17H12, catalog number 560702, 10 µl), IFNγ (BD Biosciences, clone B27, catalog number 560924, 5 µl), and TNFα (BD Biosciences, clone Mab11, catalog number 552889, 10 µl) for 20 min at 4 °C in the dark, then washed in FACS staining buffer, and fixed in 200 µl freshly prepared PBS with 0.1% paraformaldehyde (Alfa Aesar, Haverhill, MA). Uncompensated flow cytometric events were acquired on a Beckton-Dickinson LSR-II flow cytometer (BD Immunocytometry Systems, San Jose, CA) 1–24 h after fixation. BD CompBeads (Anti-Mouse IgGκ and Anti-Rat IgG/Negative Control (FBS) compensation particles, BD Biosciences) stained separately with the individual monoclonal antibodies used in the test samples, were used as a basis for electronic compensation. Samples were analyzed in one single technical replicate.

### Multi-parameter ICS data analysis

Test samples were electronically compensated for spectral spill-over of fluorochromes and gated using FlowJo version 9.4.11 software (FlowJo LLC., Ashland, OR). Gates for the expression of CD69, IFNγ, TNFα, IL-2, CD95, and CD28 on PBMCs were based on fluorescence-minus-one controls (FMO-controls) as described in ref. ^63^ and on positive and negative ICS controls. Lymphocytes were identified on forward and side scatter (FSC and SSC) characteristics after exclusion of doublets based on their FSC-area/FSC-height characteristics, exclusion of dead cells based on positivity for the Live/Dead cell discrimination fluorochrome (amine reactive) and based on determination of CD69 co-expression. To allow multifunctionality analysis, Boolean gates were calculated for activated, cytokine-secreting cells. ICS data were subjected to an exploratory positivity analysis, which utilized the method described in ref. ^64^ To identify samples responding to the RSV-FA2 peptide pool stimulation with expression of IFNγ, TNFα or IL-2 within the CD4 and CD8 T-cell subsets, a two-by-two contingency table was created to compare data from the peptide-stimulated test samples with their corresponding negative controls. Each of the tables contained four records with the number of cells positive and negative for expression of one of the detected cytokines (IFNγ, TNFα, or IL-2) in the test sample and negative control sample. To evaluate whether the number of positive cells in the stimulated sample was equal to that in the negative control, a one-sided Fisher's exact test was applied. The T-cell subset was marked as a responder to RSV-A2 peptides stimulation when the *p* value for the test sample exceeded 0.00001.

Polyfunctionality analysis was performed separately for CD4 and CD8 T cells on Boolean cytokine gates. For visualization purposes, all individual populations were subjected to background subtraction based on their corresponding negative controls and any resulting negative values were censored to 0.

### Statistical analyses

All immunological parameters (i.e., ELISA, VNA, ELISPOT, ICS) were log-transformed. Vaccine regimens were compared using two-sided tests based on analysis-of-variance, or analysis-of-variance for potentially censored values (Tobit regression). A Tukey correction was applied to account for multiple testing. *P* values < 0.05 were considered statistically significant. The correlation between assays was examined using the Spearman’s rank correlation coefficient. Since no formal lower limit of quantification was determined for ICS data, a stringent, exploratory statistical responder analysis was performed at week 14, to determine whether the antigen-specific T-cell responses after peptide stimulation differed significantly from background responses. Positive responses to RSV-F peptide stimulation within each functional T-cell population were determined per animal in comparison with the corresponding negative control, using a one-sided Fisher’s exact test with Bonferroni adjustment for multiple comparisons. All analyses were performed in R 3.3.3.^65^

### Reporting summary

Further information on research design is available in the Nature Research Reporting Summary linked to this article.

## Supplementary information

Supplemental Material

Reproducibility checklist - reporting summary

## Data Availability

The data that support the findings of this study are available from the corresponding author upon reasonable request.

## Code availability

No custom code or mathematical algorithm was used for the analysis of the data.

## References

[1] Rima B (2017). ICTV virus taxonomy profile: pneumoviridae. J. Gen. Virol..

[2] Nair H (2010). Global burden of acute lower respiratory infections due to respiratory syncytial virus in young children: a systematic review and meta-analysis. Lancet.

[3] Falsey AR, Walsh EE (2000). Respiratory syncytial virus infection in adults. Clin. Microbiol. Rev..

[4] Hall CB, Walsh EE, Long CE, Schnabel KC (1991). Immunity to and frequency of reinfection with respiratory syncytial virus. J. Infect. Dis..

[5] Falsey AR, Hennessey PA, Formica MA, Cox C, Walsh EE (2005). Respiratory syncytial virus infection in elderly and high-risk adults. N. Engl. J. Med..

[6] Han LL, Alexander JP, Anderson LJ (1999). Respiratory syncytial virus pneumonia among the elderly: an assessment of disease burden. J. Infect. Dis..

[7] Thompson WW (2003). Mortality associated with influenza and respiratory syncytial virus in the United States. JAMA.

[8] Palivizumab A (1998). Humanized respiratory syncytial virus monoclonal antibody, reduces hospitalization from respiratory syncytial virus infection in high-risk infants. Pediatrics.

[9] Committee on Infectious, D. (2009). From the American Academy of Pediatrics: Policy statements–Modified recommendations for use of palivizumab for prevention of respiratory syncytial virus infections. Pediatrics.

[10] Fulginiti VA (1969). Respiratory virus immunization. I. A field trial of two inactivated respiratory virus vaccines; an aqueous trivalent parainfluenza virus vaccine and an alum-precipitated respiratory syncytial virus vaccine. Am. J. Epidemiol..

[11] Kapikian AZ, Mitchell RH, Chanock RM, Shvedoff RA, Stewart CE (1969). An epidemiologic study of altered clinical reactivity to respiratory syncytial (RS) virus infection in children previously vaccinated with an inactivated RS virus vaccine. Am. J. Epidemiol..

[12] Connors M (1994). Enhanced pulmonary histopathology induced by respiratory syncytial virus (RSV) challenge of formalin-inactivated RSV-immunized BALB/c mice is abrogated by depletion of interleukin-4 (IL-4) and IL-10. J. Virol..

[13] Polack FP (2002). A role for immune complexes in enhanced respiratory syncytial virus disease. J. Exp. Med..

[14] Mazur NI (2018). The respiratory syncytial virus vaccine landscape: lessons from the graveyard and promising candidates. Lancet Infect. Dis..

[15] Glezen WP, Taber LH, Frank AL, Kasel JA (1986). Risk of primary infection and reinfection with respiratory syncytial virus. Am. J. Dis. Child..

[16] Kawasaki Y, Hosoya M, Katayose M, Suzuki H (2004). Role of serum neutralizing antibody in reinfection of respiratory syncytial virus. Pediatrics Int..

[17] Kurzweil V (2013). Translational sciences approach to RSV vaccine development. Expert Rev. Vaccines.

[18] Falsey AR (2008). Comparison of the safety and immunogenicity of 2 respiratory syncytial virus (rsv) vaccines–nonadjuvanted vaccine or vaccine adjuvanted with alum–given concomitantly with influenza vaccine to high-risk elderly individuals. J. Infect. Dis..

[19] Groothuis JR (1993). Prophylactic administration of respiratory syncytial virus immune globulin to high-risk infants and young children. The Respiratory Syncytial Virus Immune Globulin Study Group. N. Engl. J. Med.

[20] Piedra PA, Jewell AM, Cron SG, Atmar RL, Glezen WP (2003). Correlates of immunity to respiratory syncytial virus (RSV) associated-hospitalization: establishment of minimum protective threshold levels of serum neutralizing antibodies. Vaccine.

[21] Walsh EE, Falsey AR (2004). Humoral and mucosal immunity in protection from natural respiratory syncytial virus infection in adults. J. Infect. Dis..

[22] Ngwuta JO (2015). Prefusion F-specific antibodies determine the magnitude of RSV neutralizing activity in human sera. Sci. Transl. Med..

[23] Larranaga CL (2009). Impaired immune response in severe human lower tract respiratory infection by respiratory syncytial virus. Pediatr. Infect. Dis. J..

[24] de Bree GJ (2005). Respiratory syncytial virus-specific CD8+ memory T cell responses in elderly persons. J. Infect. Dis..

[25] Cherukuri A (2013). Adults 65 years old and older have reduced numbers of functional memory T cells to respiratory syncytial virus fusion protein. Clin. Vaccine Immunol..

[26] Roumanes D (2018). T-cell responses in adults during natural respiratory syncytial virus infection. J. Infect. Dis..

[27] Habibi MS (2015). Impaired antibody-mediated protection and defective IgA B-cell memory in experimental infection of adults with respiratory syncytial virus. Am. J. Respir. Crit. Care Med..

[28] Jozwik A (2015). RSV-specific airway resident memory CD8+ T cells and differential disease severity after experimental human infection. Nat. Commun..

[29] Barouch DH (2011). International seroepidemiology of adenovirus serotypes 5, 26, 35, and 48 in pediatric and adult populations. Vaccine.

[30] Mast TC (2010). International epidemiology of human pre-existing adenovirus (Ad) type-5, type-6, type-26 and type-36 neutralizing antibodies: correlates of high Ad5 titers and implications for potential HIV vaccine trials. Vaccine.

[31] Zahn R (2012). Ad35 and ad26 vaccine vectors induce potent and cross-reactive antibody and T-cell responses to multiple filovirus species. PLoS ONE.

[32] Salisch NC (2017). Antigen capsid-display on human adenovirus 35 via pIX fusion is a potent vaccine platform. PLoS ONE.

[33] Rodriguez A (2009). Evaluation of a prime-boost vaccine schedule with distinct adenovirus vectors against malaria in rhesus monkeys. Vaccine.

[34] Hoft DF (2012). A recombinant adenovirus expressing immunodominant TB antigens can significantly enhance BCG-induced human immunity. Vaccine.

[35] Ouedraogo A (2013). A phase 1b randomized, controlled, double-blinded dosage-escalation trial to evaluate the safety, reactogenicity and immunogenicity of an adenovirus type 35 based circumsporozoite malaria vaccine in Burkinabe healthy adults 18 to 45 years of age. PLoS ONE.

[36] Barouch DH (2013). Characterization of humoral and cellular immune responses elicited by a recombinant adenovirus serotype 26 HIV-1 Env vaccine in healthy adults (IPCAVD 001). J. Infect. Dis..

[37] Abel B (2010). The novel tuberculosis vaccine, AERAS-402, induces robust and polyfunctional CD4+ and CD8+ T cells in adults. Am. J. Respir. Crit. Care Med..

[38] Graham BS (2011). Biological challenges and technological opportunities for respiratory syncytial virus vaccine development. Immunol. Rev..

[39] Vellinga J (2014). Challenges in manufacturing adenoviral vectors for global vaccine product deployment. Hum. Gene Ther..

[40] Widjojoatmodjo MN (2015). Recombinant low-seroprevalent adenoviral vectors Ad26 and Ad35 expressing the respiratory syncytial virus (RSV) fusion protein induce protective immunity against RSV infection in cotton rats. Vaccine.

[41] Gardner MB, Luciw PA (2008). Macaque models of human infectious disease. ILAR J..

[42] De Swart RL (2002). Immunization of macaques with formalin-inactivated respiratory syncytial virus (RSV) induces interleukin-13-associated hypersensitivity to subsequent RSV infection. J. Virol..

[43] Magro M (2012). Neutralizing antibodies against the preactive form of respiratory syncytial virus fusion protein offer unique possibilities for clinical intervention. Proc. Natl Acad. Sci. USA.

[44] McLellan JS (2013). Structure-based design of a fusion glycoprotein vaccine for respiratory syncytial virus. Science.

[45] Graham BS, Modjarrad K, McLellan JS (2015). Novel antigens for RSV vaccines. Curr. Opin. Immunol..

[46] Grandin C (2015). Evidence for an intranasal immune response to human respiratory syncytial virus infection in cynomolgus macaques. J. Gen. Virol..

[47] Zhang B (2017). Protection of calves by a prefusion-stabilized bovine RSV F vaccine. NPJ Vaccines.

[48] Steff AM (2017). Pre-fusion RSV F strongly boosts pre-fusion specific neutralizing responses in cattle pre-exposed to bovine RSV. Nat. Commun..

[49] Krarup A (2015). A highly stable prefusion RSV F vaccine derived from structural analysis of the fusion mechanism. Nat. Commun..

[50] Collins PL, Melero JA (2011). Progress in understanding and controlling respiratory syncytial virus: still crazy after all these years. Virus Res..

[51] Grunwald T (2014). Novel vaccine regimen elicits strong airway immune responses and control of respiratory syncytial virus in nonhuman primates. J. Virol..

[52] Pierantoni Angiolo, Esposito Maria Luisa, Ammendola Virginia, Napolitano Federico, Grazioli Fabiana, Abbate Adele, del Sorbo Mariarosaria, Siani Loredana, D'Alise Anna Morena, Taglioni Alessandra, Perretta Gemma, Siccardi Antonio, Soprana Elisa, Panigada Maddalena, Thom Michelle, Scarselli Elisa, Folgori Antonella, Colloca Stefano, Taylor Geraldine, Cortese Riccardo, Nicosia Alfredo, Capone Stefania, Vitelli Alessandra (2015). Mucosal delivery of a vectored RSV vaccine is safe and elicits protective immunity in rodents and nonhuman primates. Molecular Therapy - Methods & Clinical Development.

[53] Wang, D. et al. A single-dose recombinant parainfluenza virus 5-vectored vaccine expressing respiratory syncytial virus (RSV) F or G protein protected cotton rats and african green monkeys from RSV challenge. *J. Virol.* **91**. 10.1128/JVI.00066-17 (2017). 10.1128/JVI.00066-17 PMC5432884 28298602

[54] Patton K (2015). Enhanced immunogenicity of a respiratory syncytial virus (RSV) F subunit vaccine formulated with the adjuvant GLA-SE in cynomolgus macaques. Vaccine.

[55] Stewart VA (2007). Priming with an adenovirus 35-circumsporozoite protein (CS) vaccine followed by RTS,S/AS01B boosting significantly improves immunogenicity to Plasmodium falciparum CS compared to that with either malaria vaccine alone. Infect. Immun..

[56] Kakuk TJ (1993). A human respiratory syncytial virus (RSV) primate model of enhanced pulmonary pathology induced with a formalin-inactivated RSV vaccine but not a recombinant FG subunit vaccine. J. Infect. Dis..

[57] Barouch DH (2018). Evaluation of a mosaic HIV-1 vaccine in a multicentre, randomised, double-blind, placebo-controlled, phase 1/2a clinical trial (APPROACH) and in rhesus monkeys (NHP 13-19). Lancet.

[58] Maizel JV, White DO, Scharff MD (1968). The polypeptides of adenovirus. I. Evidence for multiple protein components in the virion and a comparison of types 2, 7A, and 12. Virology.

[59] Fallaux FJ (1996). Characterization of 911: a new helper cell line for the titration and propagation of early region 1-deleted adenoviral vectors. Hum. Gene Ther..

[60] Graham FL, Smiley J, Russell WC, Nairn R (1977). Characteristics of a human cell line transformed by DNA from human adenovirus type 5. J. Gen. Virol..

[61] Sprangers MC (2003). Quantifying adenovirus-neutralizing antibodies by luciferase transgene detection: addressing preexisting immunity to vaccine and gene therapy vectors. J. Clin. Microbiol..

[62] Gauduin MC (2006). Intracellular cytokine staining for the characterization and quantitation of antigen-specific T lymphocyte responses. Methods.

[63] Roederer M (2002). Compensation in flow cytometry. Curr. Protoc. Cytom..

[64] Wecker M (2012). Phase I safety and immunogenicity evaluations of an alphavirus replicon HIV-1 subtype C gag vaccine in healthy HIV-1-uninfected adults. Clin. Vaccin. Immunol..

[65] Team, R. D. C. R. (2011). A Language and Environment for Statistical Computing.

