# Supplementary material for: Adenovectors encoding RSV-F protein induce durable and mucosal immunity in macaques after two intramuscular administrations
Source: NPJ Vaccines. 2019 Dec 20;4:54. doi: 10.1038/s41541-019-0150-4 (PMC6925274; doi:10.1038/s41541-019-0150-4)
Supplement: Supplementary file 1 — Supplemental Material [file 41541_2019_150_MOESM1_ESM.pdf]

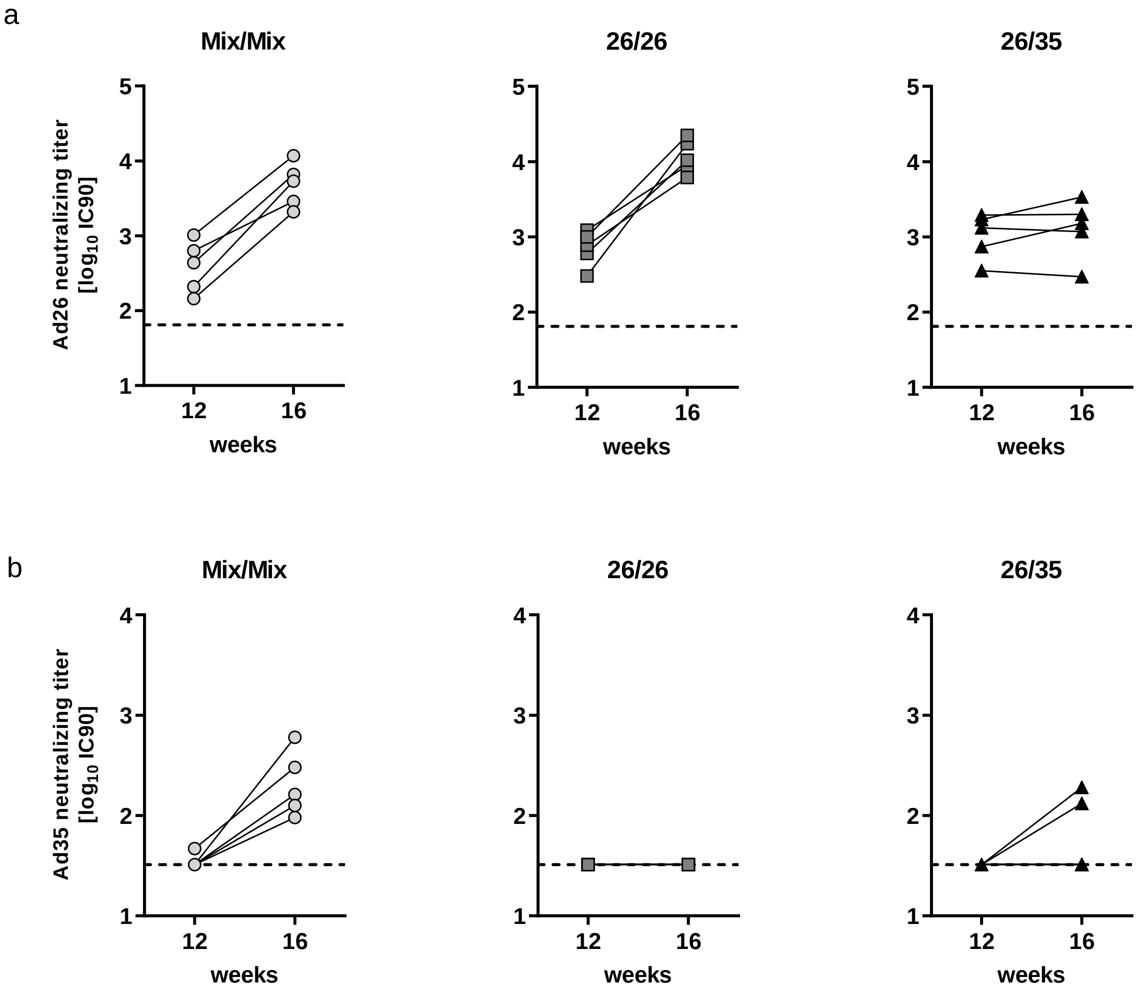


**Supplementary Figure 1: Anti-vector responses**. Ad26 (a) and Ad35 (b) neutralizing antibody titers at time point of boost (week 12) and 4 weeks post-boost (week 16) were determined using an Ad26- or Ad35-based VNA in sera obtained from animals in the indicated groups. Each symbol represents one animal, dotted line depicts lower limit of detection (LLoD; $log_2$ lowest serum dilution in the assay, 1/64 for Ad26 and 1/32 for Ad35). N=5 animals per group.

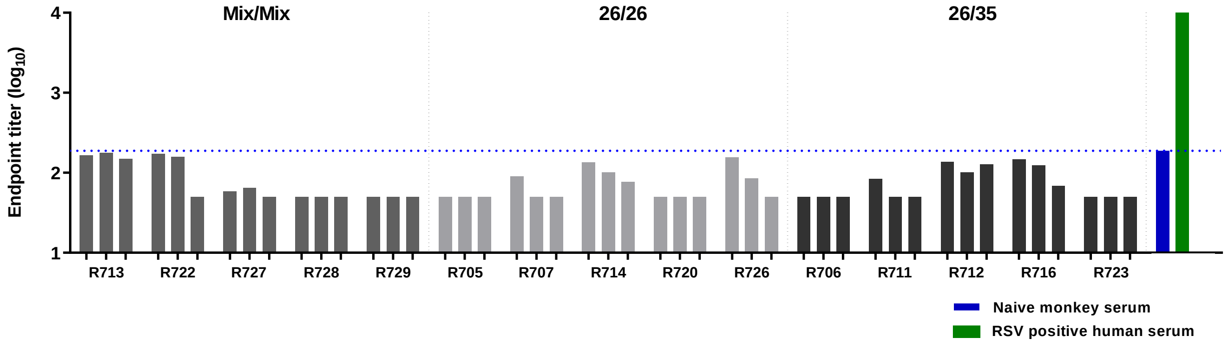


**Supplementary Figure 2: RSV-G specific IgG titers.** RSV-G specific binding antibody titers in serum samples of individual animals taken at weeks 25, 32, and 65 post prime (from left to right) were assessed by ELISA and are represented by vertical bars. Serum from a naïve, female macaque purchased at the Biomedical Primate Research Center, Rijswijk, The Netherlands (blue bar) and from an RSV-positive human purchased at Seralab, UK (now BioIVT; green bar) were included as negative and positive controls, respectively. The blue dotted line represents the titer level of the naive NHP serum, for orientation.

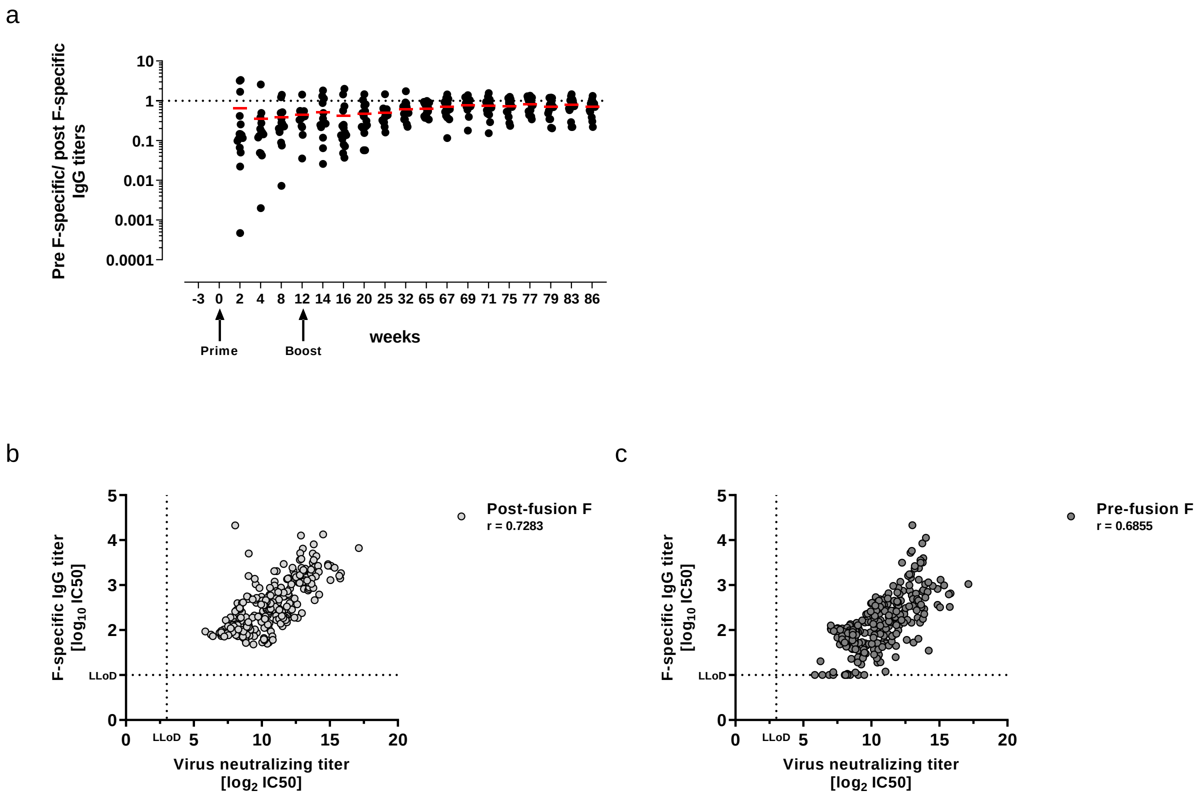


**Supplementary Figure 3: Ratios and correlations of RSV-F specific antibody responses.** a: Ratio of pre-F and post-F specific RSV-F antibody titers in individual samples, pooled across all three regimens, at all time points after prime (weeks 2-86; n=15 per time point). Shown is the ratio of linear pre-F-specific IgG titers divided by post-F-specific titers per individual animal, for each time point. Bars depict arithmetic group means. B and C: Correlation of RSV A2 neutralizing capacity with post-F specific (B) and post-F specific (C) ELISA titers, pooled across all three regimens, at all time points after prime (weeks 2-86). Each symbol represents one animal (n=270 data points). LLoD = lower limit of detection (log10 lowest serum dilution in the pre-F and post-F specific assay, 1/10 and log2 lowest serum dilution in in the virus neutralization assay, 1/8). Spearman correlation coefficients (r) were calculated.

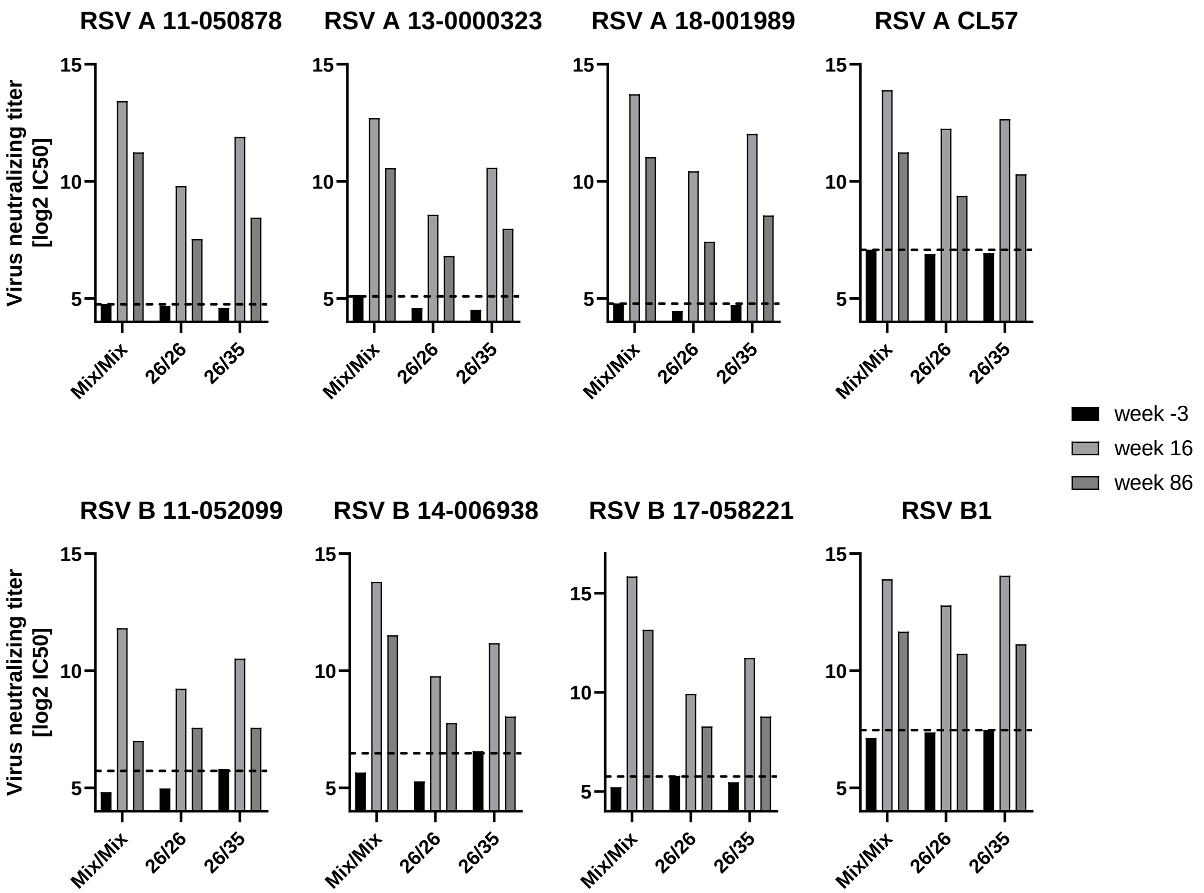


**Supplementary Figure 4: RSV A and B neutralization titers.** Titers of neutralizing antibodies targeting RSV A strains A 11-050878, A 13-0000323, A 18-0011989, A CL57 and RSV B strains B 11-052099, B 14-006938, B 17-058221, and B1 were determined in sera pooled by treatment group at weeks -3, 16, and 86. Shown are log2 inverse IC50 values per timepoint and treatment group (mean of two technical replicates); the dotted lines indicate the limit of background ($log_2$ 95$^{th}$ percentile of mean serum titers in all samples at week -3).

a

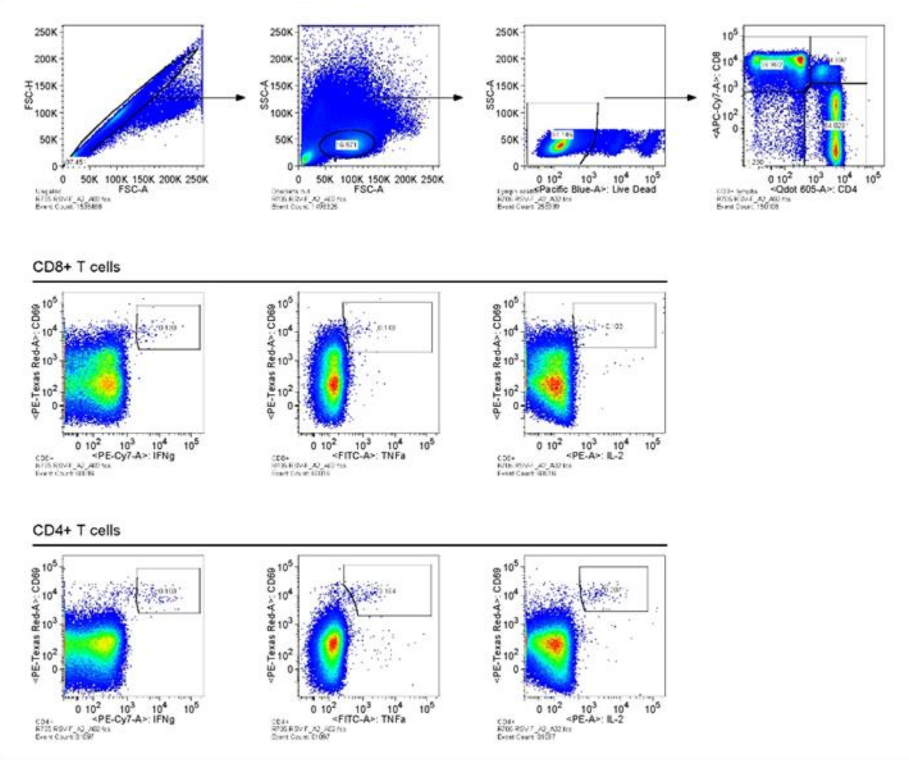
FSC-H
FSC-A
SSC-A
FSC-A
SSC-A
<Pacific Blue-A>: Live Dead
<APC-Cy7-A>: CD8
<Qdot 605-A>: CD4
CD8+ T cells
<PE-Texas Red-A>: CD69
<PE-Cy7-A>: IFNg
<FITC-A>: TNFa
<PE-A>: IL-2
CD4+ T cells
<PE-Texas Red-A>: CD69
<PE-Cy7-A>: IFNg
<FITC-A>: TNFa
<PE-A>: IL-2

b

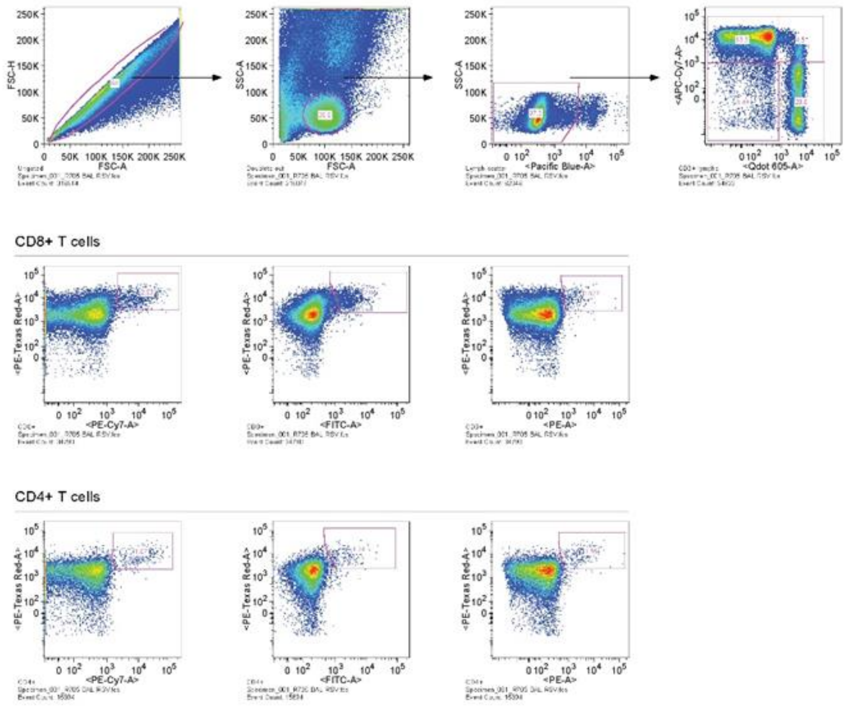
FSC-H
FSC-A
SSC-A
FSC-A
SSC-A
<Pacific Blue-A>
<APC-Cy7-A>
<Qdot 605-A>
CD8+ T cells
<PE-Texas Red-A>
<PE-Cy7-A>
<FITC-A>
<PE-A>
CD4+ T cells
<PE-Texas Red-A>
<PE-Cy7-A>
<FITC-A>
<PE-A>

**Supplementary Figure 5: Gating strategies used to identify RSV-F specific T cells in PBMC and BAL.** (A) Gating strategy used to determine RSV-FA2-specific, IFNγ, TNFα, and IL-2 secreting CD4+ T-cells in freshly isolated PBMCs by multiparametric ICS and polychromatic flow cytometry shown in Figure 4 a) – d). (B) Gating strategy used to determine RSV-FA2-specific, IFNγ, TNFα, and IL-2 secreting CD8+ and CD4+ T-cells in freshly isolated BAL cells by multiparametric ICS and polychromatic flow cytometry shown in Figure 5 a) – h).Exemplified on representative samples from animal R705 (26/26 group): PBMCs at week 14 (A) or BAL cells at week 16 (B), after stimulation with RSV-F peptides.

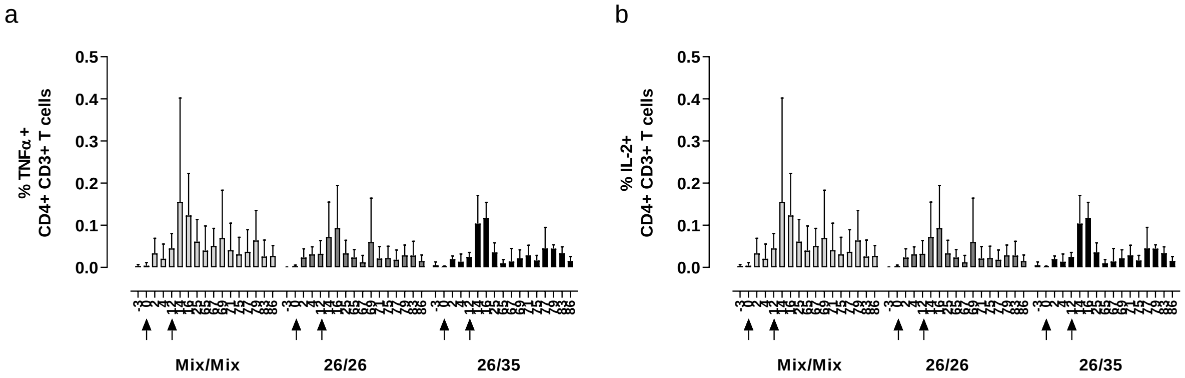


**Supplementary Figure 6: RSV-F specific TNFα and IL-2 ICS responses in PBMC.** RSV-F specific TNFα (A), and IL-2 (B) responses were determined in peripheral CD4+ T-cells using polychromatic flow cytometry after stimulation with a pool of 15-mer peptides overlapping by 11 amino acids, covering the RSV-F protein sequence. Shown are arithmetic group means with upper 95% confidence limit (upper whisker) over time, of individually background subtracted values (n=5). Animals received a homologous prime-boost with the Ad26/Ad35 mix (light grey), a homologous prime-boost with Ad26 (dark grey), or a heterologous prime-boost with Ad26 and Ad35 (black). Arrows indicate immunization time points.

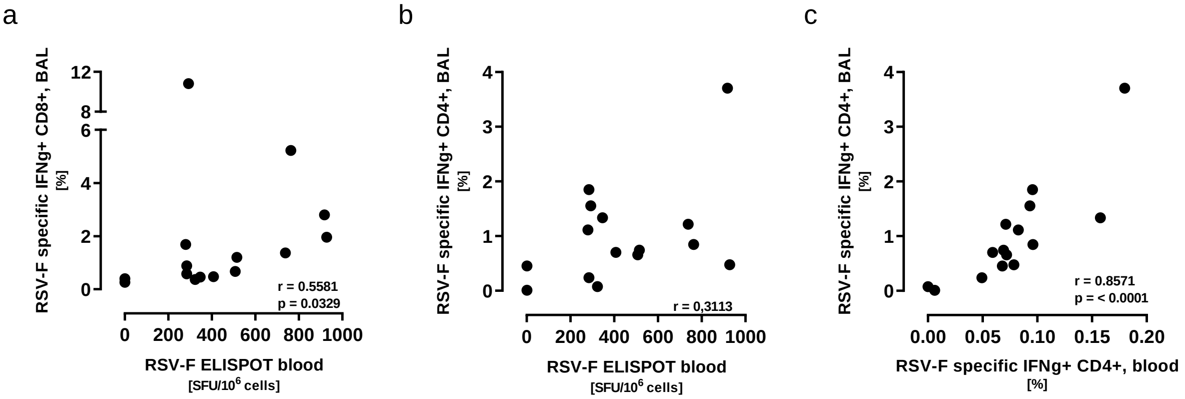


**Supplementary Figure 7: Correlation of RSV-F specific T-cell responses in the blood and in the lung.**

Correlation of RSV-F specific IFNγ ELISPOT responses in the blood with IFNγ+ CD8+ T-cell (A) or IFNγ+ CD4+ T-cell (B) responses in the lung, and of IFNγ+CD4+ T-cell responses in the blood and lung (C), at week 16 (4 weeks post-boost). Data for all analyses were pooled across all three regimens. (A and B) On the x-axis, IFNγ ELISPOT responses measured in PBMCs, on the y-axis IFNγ+ CD8+ T-cell responses (A) or CD4+ T-cells (B) measured in BAL cells by ICS, after stimulation with a pool of 15-mer peptides overlapping by 11 amino acids, covering the RSV-F protein sequence. SFU = spot forming units. (C) On the x-axis, RSV-F specific IFNγ+CD4+ T-cell responses measured in PBMCs, on the y-axis RSV-F specific IFNγ+CD4+T-cell responses measured in BAL using ICS, as described above. Values were individually background subtracted, each symbol represents one animal (n=15). A Spearman correlation coefficient (*r*) was calculated for each correlation.
